# LTBR acts as a novel immune checkpoint of tumor‐associated macrophages for cancer immunotherapy

**DOI:** 10.1002/imt2.233

**Published:** 2024-09-04

**Authors:** Liang Wang, Jieyi Fan, Sifan Wu, Shilin Cheng, Junlong Zhao, Fan Fan, Chunchen Gao, Rong Qiao, Qiqi Sheng, Yiyang Hu, Yong Zhang, Pengjun Liu, Zhe Jiao, Tiaoxia Wei, Jie Lei, Yan Chen, Hongyan Qin

**Affiliations:** ^1^ State Key Laboratory of Holistic Integrative Management of Gastrointestinal Cancers and Department of Medical Genetics and Developmental Biology Fourth Military Medical University Xi'an China; ^2^ Department of Aerospace Medicine Fourth Military Medical University Xi'an China; ^3^ Department of Clinical Oncology, Xijing Hospital Fourth Military Medical University Xi'an China; ^4^ Department of Pulmonary Medicine, Xijing Hospital Fourth Military Medical University Xi'an China; ^5^ Department of Thoracic Surgery, Tangdu Hospital Fourth Military Medical University Xi'an China

**Keywords:** immune checkpoint, LTBR, myeloid derived suppressor cells, CD8^+^ T cells, tumor‐associated macrophages

## Abstract

Tumor‐associated macrophages (TAMs) greatly contribute to immune checkpoint inhibitor (ICI) resistance of cancer. However, its underlying mechanisms and whether TAMs can be promising targets to overcome ICI resistance remain to be unveiled. Through integrative analysis of immune multiomics data and single‐cell RNA‐seq data (iMOS) in lung adenocarcinoma (LUAD), lymphotoxin β receptor (*LTBR*) is identified as a potential immune checkpoint of TAMs, whose high expression, duplication, and low methylation are correlated with unfavorable prognosis. Immunofluorescence staining shows that the infiltration of LTBR^+^ TAMs is associated with LUAD stages, immunotherapy failure, and poor prognosis. Mechanistically, LTΒR maintains immunosuppressive activity and M2 phenotype of TAMs by noncanonical nuclear factor kappa B and Wnt/β‐catenin signaling pathways. Macrophage‐specific knockout of *LTBR* hinders tumor growth and prolongs survival time via blocking TAM immunosuppressive activity and M2 phenotype. Moreover, TAM‐targeted delivery of *LTΒR* small interfering RNA improves the therapeutic effect of ICI via reversing TAM‐mediated immunosuppression, such as boosting cytotoxic CD8^+^ T cells and inhibiting granulocytic myeloid‐derived suppressor cells infiltration. Taken together, we bring forth an immune checkpoint discovery pipeline iMOS, identify LTBR as a novel immune checkpoint of TAMs, and propose a new immunotherapy strategy by targeting LTBR^+^ TAMs.

## Highlights

- Integrative analysis of immune multiomics data and single‐cell RNA‐seq data pipeline is developed and finds lymphotoxin β receptor (*LTBR*) expression specific in tumor‐associated macrophages (TAMs).
- *LTΒR* ^+^ TAMs correlate with lung adenocarcinoma stages, immunotherapy resistance, and prognosis.
- *LTΒR* maintains TAM immunosuppressive activity and M2 phenotype by noncanonical nuclear factor kappa B signaling and Wnt/β‐catenin signaling.
- Disruption of *LTΒR* in TAMs enhances the therapeutic effect of cancer immunotherapy.

## Graphical abstract

Through integrative analysis of immune multiomics data and single‐cell RNA‐seq data, this study identifies lymphotoxin β receptor (*LTBR*) as a potential immune checkpoint of tumor‐associated macrophages (TAMs). LTBR^+^ TAMs are associated with lung adenocarcinoma stages, immunotherapy failure, and poor prognosis. Mechanistically, LTΒR maintains TAM immunosuppressive activity and M2 phenotype by noncanonical nuclear factor kappa B and Wnt/β‐catenin signaling pathways. Disruption of *LTΒR* in TAMs enhances the therapeutic effect of cancer immunotherapy.

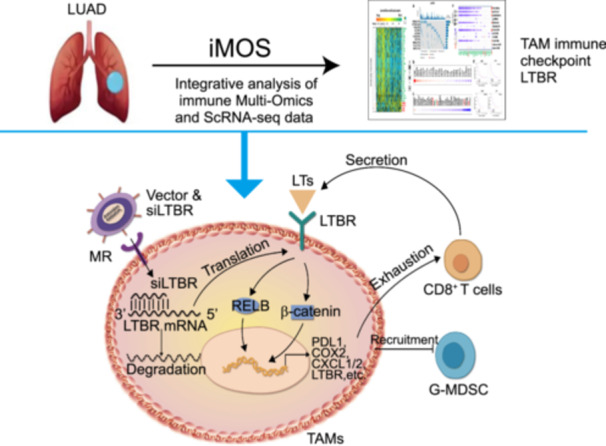

## INTRODUCTION

Lung adenocarcinoma (LUAD) occupies over 40% of lung cancer incidence, which has been the leading cause of cancer‐related mortality [1]. Accumulating evidence and our previous studies have reported that immune checkpoint inhibitors (ICIs) significantly improved the event‐free survival and pathologic complete response in lung cancer patients [2, 3], but the overall response rate of ICI is only 6.3%–26% [4], highlighting the urgency to reveal underlying mechanisms and develop novel ICI. Tumor immune microenvironment (TIM) plays a vital role in tumor initiation, progression, and immunotherapy resistance [5, 6]. Previous multiomics analysis mostly focuses on the oncogenic drivers [7, 8, 9], but few attention is paid to the multiomics feature of TIM.

Tumor‐associated macrophages (TAMs) are the major cell population of TIM, which not only nourish tumor cells but also contribute to tumor immunosuppressive microenvironment (TISM), including exhausting cytotoxic CD8^+^ T cells and recruiting immunosuppressive cells, like myeloid‐derived suppressor cells (MDSCs), regulatory T cells (Tregs), and so on [10]. Recent evidence shows that TAMs greatly contribute to ICI resistance, including pembrolizumab, avelumab, and ipilimumab [11, 12]. These studies indicate that targeting TAMs might be a promising strategy for tumor immunotherapy.

As a member of the tumor necrosis factor receptor superfamily, lymphotoxin β receptor (*LTΒR*) is constitutively expressed on stromal cells and myeloid lineage cells but not on T or B lymphocytes [13]. *LTBR* is activated by two ligands: the lymphotoxin heterotrimer LTα1β2 expressed on activated T, B, and NK cells [14] or *LIGHT* homotrimer mostly expressed on activated T cells [15, 16]. Recent study shows that *LTΒR* is involved in the differentiation of CD169^+^ macrophages in lymph node and spleen [17]. In an inflammatory study, Wimmer et al. find that *LTBR* activation of macrophages by T cell‐derived LTα1β2 acts as a counterregulatory signal against exacerbating inflammatory reaction [18], indicating the immunosuppressive role of *LTBR* in macrophages. However, whether *LTBR* activation in TAMs contributes to TISM formation and its underlying mechanism need to be further investigated.

**Figure 1:**
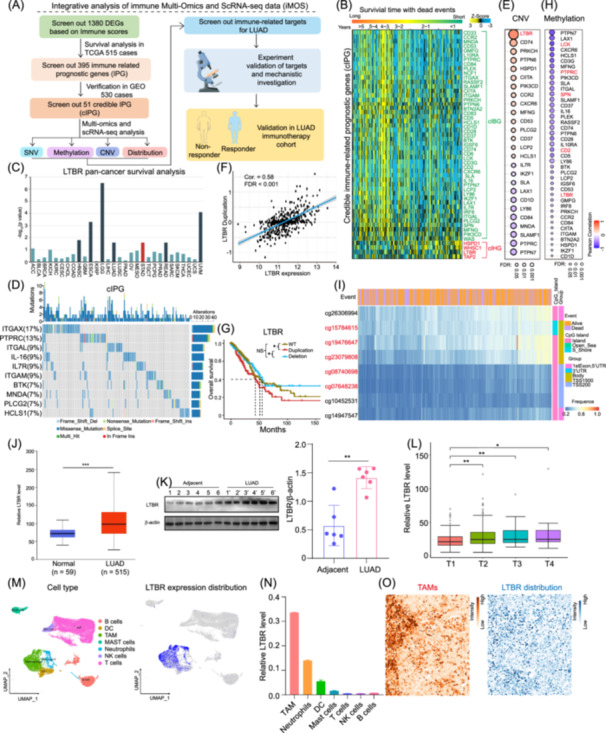
Integrative analysis of immune multiomics data and single‐cell RNA (scRNA)‐seq data (iMOS) identifies lymphotoxin *β* receptor (*LTBR*) as a potential immune checkpoint of tumor‐associated macrophages (TAMs). (A) The workflow of iMOS was displayed. (B) Based on lung adenocarcinoma (LUAD) cohorts from GEO database, credible immune‐related prognostic genes (cIPG) were screened out, including credible immune‐related beneficial genes (cIBG) and harmful genes (cIHG), *p* < 0.05 by the log‐rank test. Heatmaps showed cIPG expressions and survival times of LUAD cases. (C) The histogram showed pan‐cancer survival analysis of *LTBR*, dark blue column indicating that higher expression of the gene correlates with shorter survival (*p* < 0.05), red column indicating that higher expression of the gene correlates with longer survival (*p* < 0.05), while light blue indicating no significance by log‐rank test. (D) Waterfall plot presented the mutation distribution of the top 10 mutated genes from cIPG in LUAD. (E) Bubble plot represented the profile between the expression and copy number variation (CNV) level of cIPG. (F) The association between *LTBR* expression and its duplication was tested via Pearson correlation analysis. (G) Kaplan–Meier plot showed the duplication of *LTBR* was associated with poor survival in LUAD. (H) Bubble plot represented the profile between the expression and methylation level of cIPG. (I) The heatmap displayed the binding frequence and location of eight methylation probes in LUAD patients. (J) The relative level of *LTBR* in primary LUAD tissues (*n* = 515) and normal lung tissues (*n* = 59) was analyzed via The Cancer Genome Atlas (TCGA) database. (K) The relative protein level of LTΒR in LUAD and adjacent tissues was measured via western blot analysis (*n* = 6). (L) The relative level of *LTBR* in different T stages was analyzed via TCGA database. (M and N) By analyzing human LUAD scRNA‐seq data, Uniform Manifold Approximation and Projection plots (M) and histogram (N) showed the expression distribution of *LTBR* among immune cells. (O) Spatial scRNA‐seq analysis showed the spatial colocalization between TAMs and *LTBR* in LUAD. Data are shown as mean ± SEM. *p < 0.05; **p < 0.01; ***p < 0.001 using unpaired Student's *t* test (J, K) or one‐way analysis of variance with Tukey's multiple comparison test (L). DC, dendritic cells; GEO, genomics expression omnibus; IL, interleukin; NK cells, natural killer cells; SNV, single‐nucleotide variation.

In this study, we develop an immune checkpoint discovery pipeline through integrative analysis of immune multiomics data and single‐cell RNA (scRNA)‐seq data (iMOS) (Figure 1A) and propose that *LTΒR* can be a potential immune checkpoint of TAMs, whose high expression, duplication, and low methylation are corelated with LUAD unfavorable prognosis. Immunofluorescent staining of LUAD tissue microarray shows that the infiltration of *LTBR* ^+^ TAMs is associated with LUAD stages, immunotherapy failure, and poor prognosis. Mechanistically, *LTΒR* maintains TAM immunosuppressive activity and M2 phenotype by noncanonical nuclear factor kappa B (NF‐κB) signaling and Wnt/β‐catenin signaling. Macrophage‐specific knockout of *LTBR* hinders tumor growth and prolongs survival time via blocking TAM immunosuppressive activity and M2 phenotype. Moreover, TAM‐targeted delivery of *LTΒR* small interfering RNA (siRNA) improves the therapeutic effect of ICI via reversing TAM‐mediated immunosuppression, such as boosting cytotoxic CD8^+^ T cells and inhibiting granulocytic myeloid‐derived suppressor cell (G‐MDSC) infiltration. Collectively, our study develops an immune checkpoint discovery pipeline iMOS and identifies *LTBR* as a novel immune checkpoint of TAMs that promotes CD8^+^ T cell exhaustion and G‐MDSC recruitment through noncanonical NF‐κB signaling and Wnt/β‐catenin signaling and proposes a new cancer immunotherapy strategy by targeting *LTBR* on TAMs.

## RESULTS

### Screening out immune‐related genes in LUAD by immune scores

The clinical information and gene expression data of 522 LUAD cases were downloaded from the cancer genome atlas (TCGA) database. The clinical characters in this study are shown in Table S1. Only 515 cases that had both clinical information and gene expression data were further analyzed. Based on the gene expression data of LUAD cases, estimation of stromal and immune cells in malignant tumours using expression data (ESTIMATE) [19] algorithm was performed to obtain the immune scores of LUAD patients. Immune scores were associated with pathological grades, TNM stages, and overall survival (OS) in LUAD patients (Figure S1A–E), which could be adapted to predict the tumor malignancy and prognosis of LUAD patients.

Based on immune scores, we screened out immune‐related genes to uncover immune determinants during LUAD progression (Figure S1F). First, 515 LUAD cases from TCGA database were divided into two groups with high and low immune scores. Then, 1380 differentially expressed genes (DEGs) (*p* < 0.05, fold change [FC] > 1.5) were identified (Figure S1F, Table S2). Moreover, gene ontology (GO) analysis showed that these DEGs were involved in lymphocyte activation, regulation of cytokine production, leukocyte activation involved in immune response, and so on (Figure S1G). Through Kyoto Encyclopedia of Genes and Genomes (KEGG) analysis, the top three enriched pathways of these DEGs included macrophage differentiation, interleukin‐12 (*IL12*) pathway, and cytokine‐cytokine receptor interaction (Figure S1H). These results manifested the effectiveness of immune scores in screening out immune‐related DEGs.

### Screening out credible immune‐related prognostic genes (IPGs) in LUAD cohorts

To investigate whether the expression of immune‐related DEGs was associated with LUAD OS, we performed the survival analysis of 1380 immune‐related DEGs. Among them, 395 IPGs were significantly associated with LUAD OS (Figure S2A, Table S3). Moreover, IPGs were divided into 343 immune‐related beneficial genes (IBGs, higher expression associated with longer survival) and 52 immune‐related harmful genes (IHG, higher expression associated with shorter survival) (Figure S2A). Interestingly, we found that the relative average expression level of IBG increased with survival time extending, while that of IHG decreased correspondingly (Figure S2B,C). Furthermore, GO analysis showed that 395 IPG were involved in lymphocyte activation, immune response, leukocyte activation, and so on (Figure S2D,E). The top three KEGG‐enriched terms of these IPG contained T cell receptor (TCR) signaling pathway, cell adhesion molecules, and macrophage differentiation (Figure S2F,G). These results indicated that 395 IPG might affect LUAD prognosis through regulating immune processes and relevant signaling pathways.

To explore whether these IPGs from TCGA cohort also own prognostic significance in other LUAD cohorts, we retrieved four cohorts containing 530 LUAD cases, in PubMed, Scopus, MEDLINE, and EMBASE databases, and their related data were downloaded from the genomics expression omnibus (GEO) database (GSE8894 [20], GSE13213 [21], GSE43767 [22], and GSE68465 [23]). Among 395 IPGs, 51 genes were validated to be significantly associated with the clinical outcomes of LUAD cases from GEO database (*p* < 0.05) (Table S4), suggesting their credibility in different LUAD cohorts, which were then named as credible IPGs (cIPGs). The heatmap visually showed the correlation between the cIPG expression and survival time of LUAD cases. Herein, cIPGs were then divided into 47 credible IBGs (cIBGs) and four credible IHGs (cIHGs) (Figure 1B). Importantly, we found that the relative expression level of cIBG increased, while that of cIHG decreased with survival time extending (Figure S2H,I). Moreover, the top three GO‐enriched terms of these cIPG were leukocyte activation, regulation of cell activation, and positive regulation of immune response (Figure S2J,K). KEGG analysis showed that these cIPGs were involved in primary immunodeficiency, hematopoietic cell lineage, and TCR signaling pathway (Figure S2L,M). Notably, pan‐cancer survival analysis showed that four cIHGs, including *LTΒR*, heat shock protein family D (Hsp60) member 1, Wolf‐Hirschhorn syndrome candidate 1, and transporter 2 ATP binding cassette subfamily B member, also correlated with poor prognosis in other types of cancers, indicating their essential roles in tumor progression (Figures 1C and S2N,O).

### iMOS identifies *LTBR* as a potential immune checkpoint of TAMs

Based on the above findings, we further performed iMOS and investigated whether single‐nucleotide variation (SNV), copy number variation (CNV), and methylation of cIPG would affect LUAD prognosis. First, SNV analysis showed that 49 genes of cIPGs had SNV alterations (Table S5). Oncoplot waterfall plot delineated top 10 SNV frequency genes of cIPG (Figure 1D). The mutation of *IL16* and hematopoietic cell‐specific Lyn substrate 1 was associated with poor survival of LUAD patients (Figure S3A,B). The CNV of 26 genes of cIPG was significantly (false discovery rate [FDR] < 0.05) correlated with their expression, in which the correlation between *LTBR* duplication and its expression was the highest (Figure 1E,F, Table S6). Notably, the *LTΒR* duplication was significantly associated with the unfavorable prognosis of LUAD, implying that the *LTΒR* duplication might influence LUAD progression (Figure 1G). Methylation analysis showed that the methylation level of 41 genes among cIPGs was negatively associated with their messenger RNA (mRNA) expression (Figure 1H). Furthermore, five methylation probes (cg15784615, cg19476647, cg23079808, cg08740698, and cg07648238), binding to the transcriptional start site and gene body of *LTΒR*, identified that low methylation of *LTΒR* was correlated with unfavorable prognosis, suggesting the vital role of *LTΒR* methylation in LUAD progression (Figures 1I and S3C, Table S7). Thus, multiomics analysis revealed that the duplication and low methylation of *LTBR* were corelated with the poor survival, suggesting the involvement of *LTBR* in LUAD progression.

To further explore the potential role of *LTBR* in LUAD, we first investigated *LTBR* expression in normal lung and LUAD tissues from TCGA database, and the results showed higher expression level of *LTBR* in LUAD tissues, which was in accordance with its ligand LTα1β2 rather than *LIGHT*, suggesting that the LTα1β2/*LTBR* signal was involved in LUAD development (Figures 1J and S3D). Meanwhile, the protein level of LTBR was significantly higher in clinical resected LUAD than that in adjacent tissues (Figure 1K). Furthermore, the expression of *LTBR* exhibited an ascending trend as LUAD pathological grade, T stage, and N stage (Figures 1L and S3E). Second, we took advantage of human LUAD and normal lung tissue scRNA‐seq data and found that the highest mRNA level of *LTΒR* was observed in TAMs rather than other tumor‐infiltrated immune cells and even the macrophages of normal lung tissues (Figures 1M,N and S4A,B), while its ligand LTα1β2 was mainly expressed by lymphoid cells, including T, B, and NK cells (Figure S4C), suggesting potential crosstalk of LTα1β2/*LTBR* between lymphoid cells and TAMs. Consistently, the protein level of LTΒR in TAMs was also the highest among all immune cells by fluorescence‐activated cell sorter (FACS) assay with murine lung cancer tissue (Figure S4D). The specific expression of *LTΒR* in TAMs was further supported by reanalyzing LUAD spatial scRNA‐seq data (Figures 1O and S4E). Overall, our data indicated that *LTBR* might serve as a potential immune checkpoint of TAMs.

### *LTBR* ^+^ TAMs are associated with LUAD stages, immunotherapy failure, and clinical prognosis

**Figure 2:**
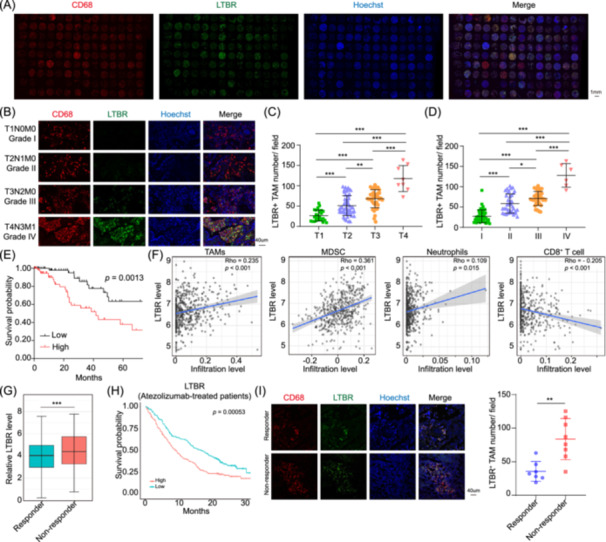
Lymphotoxin *β* receptor (LTBR)^+^ tumor‐associated macrophages (TAMs) are associated with lung adenocarcinoma (LUAD) stages, immunotherapy failure, and clinical prognosis. (A) The immunofluorescence staining of LUAD tissue microarray was displayed. (B) The representative immunofluorescence staining of LUAD tissues from (A) was showed. (C, D) The number of *LTΒR* ^+^ TAMs in LUAD patients with different tumor stages (C) and pathological grades (D) was compared. (E) LUAD patients from (A) were divided into two groups: high infiltration of *LTΒR* ^+^ TAMs (high) and low infiltration of *LTΒR* ^+^ TAMs (low), and the correlation between LUAD survival and the infiltration of *LTΒR* ^+^ TAMs was analyzed by log‐rank test. (F) The correlation between *LTBR* expression and the infiltration of indicated immune cells was analyzed by using TIMER2.0 website. (G) In OAK and POPLAR immunotherapy cohorts, the relative level of *LTBR* in patients, with responses or without responses to atezolizumab treatment, was compared. (H) In OAK and POPLAR immunotherapy cohorts, patients administrated by atezolizumab were divided into two groups by *LTBR* expression: high expression (high) and low expression (low) group, whose survival analysis is analyzed by Kaplan–Meier method and tested by the log‐rank test. (I) Immunofluorescence staining was used to compare the number of *LTΒR* ^+^ TAMs in the immunotherapy responders and nonresponders of TD‐FOREKNOW cohort. Data are shown as mean ± SEM. *p < 0.05; **p < 0.01; ***p < 0.001 using one‐way analysis of variance with Tukey's multiple comparison test (C, D) or unpaired Student's *t* test (I).

Due to *LTBR* mostly expressed in TAMs, we further investigated the association between *LTΒR* ^+^ TAMs and LUAD stages. Immunofluorescence staining of LUAD tissue microarray with 126 clinical cases showed that the infiltration of *LTΒR* ^+^ TAMs increased along with the malignancy of LUAD (Figure 2A–D). Importantly, the infiltration of *LTΒR* ^+^ TAMs could be used to predict the OS of LUAD patients (Figure 2E). These results indicated that *LTBR* ^+^ TAMs are associated with LUAD stages and clinical prognosis.

Next, to address the role of *LTBR* ^+^ TAMs in TIM, we analyzed 522 LUAD patients from TCGA database and found that the *LTΒR* expression level was positively correlated with the infiltration of TAMs, MDSC, and neutrophils, while negatively correlated with CD8^+^ T cell infiltration, but not significantly correlated with the other immune cells (Figures 2F and S5A–F). The above evidence implied that *LTBR* ^+^ TAMs might be involved in the formation of TISM. As we know, TAMs can suppress T cell recruitment and activation and, therefore, contribute to immunotherapy resistance [24, 25]. Based on these reports, we analyzed RNA‐seq data from two randomized clinical trial (RCT) cohorts (OAK, *n* = 699 and POPLAR, *n* = 192), which comprised the largest transcriptomic database of non‐small cell lung cancer patients treated with ICI. In the patients treated with atezolizumab, the expression of *LTΒR* in nonresponders was much higher than that in responders (Figure 2G). Moreover, atezolizumab‐treated patients with higher *LTΒR* expression had a worse prognosis than those with lower *LTΒR* expression (Figure 2H). Last but not least, our previous TD‐FOREKNOW phase 2 multicenter RCT cohort [3] (ClinicalTrials.gov: NCT04338620) recruited 15 resectable stages IIIA or IIIB (T3N2) LUAD patients, who were then treated with camrelizumab plus chemotherapy. Among them, seven patients, achieving a major pathologic response (MPR, defined as the presence of ≤10% viable tumor cells in the resected primary tumor specimen and sampled regional lymph nodes), were considered as responders to camrelizumab plus chemotherapy. Eight patients, not achieving MPR, were considered as nonresponders. Using the surgically resected LUAD tissues of the above patients, immunofluorescence analysis showed that the infiltration of *LTΒR* ^+^ TAMs in the LUAD tissues of nonresponders was much more than that in responders (Figure 2I). Taken together, these results indicated that *LTΒR* could be utilized to predict the immunotherapy response and clinical prognosis.

### *LTΒR* contributes to TAM‐mediated immunosuppression of CD8^+^ T cells and recruitment of G‐MDSC

**Figure 3:**
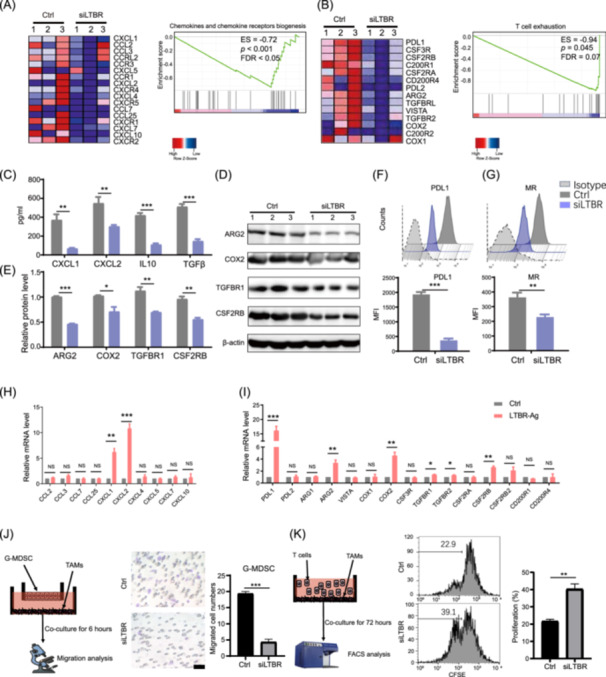
Lymphotoxin *β* receptor (LTBR) contributes to tumor‐associated macrophage (TAM) immunosuppressive activity and M2 phenotype. (A, B) RNA‐sequencing data of TAMs treated with control small interfering RNA (siRNA) (Ctrl) and *LTBR* siRNA (si*LTBR*) was utilized for gene set enrichment analysis of chemokines and chemokine receptors biogenesis (A) and T cell exhaustion (B). And the heatmaps showed the differentially expressed genes (*p* < 0.05 and fold change > 1.5) between Ctrl and si*LTBR* group. (C) The supernatant concentration of indicated chemokines and cytokines from TAMs treated with Ctrl and si*LTBR* was analyzed via enzyme‐linked immunosorbent assay array. (D) After transfection of si*LTBR* or Ctrl in TAMs, the protein level of ARG2, COX2, TGFβR1, and CSF2RB was tested by western blot analysis (*n* = 3). β‐actin was used as loading control. (E) The relative protein levels in (D) were compared (*n* = 3). (F, G) The mean fluorescence intensity of programmed cell death 1 ligand 1 (PDL1) (F) and mannose receptor (MR) (G) in TAMs treated with Ctrl and si*LTBR* was measured by a fluorescence‐activated cell sorter (FACS) (*n* = 3). (H, I) After activation of LTΒR in TAMs by agonistic LTΒR antibodies, the expression of genes involved in chemokines and chemokine receptor biogenesis (H) and T cell exhaustion (I) was measured by quantitative reverse transcription polymerase chain reaction (*n* = 3). (J) TAMs and sorted granulocytic myeloid‐derived suppressor cell (G‐MDSC) were co‐cultured in the transwell culture system, and the migration of G‐MDSC was observed by microscopy (*n* = 3). (K) TAMs transfected with si*LTBR* or Ctrl were co‐cultured with CFSE‐labelled allogeneic T cells for 72 h. The proliferation of T cells was determined by FACS (*n* = 3). Data are shown as mean ± SEM. *p < 0.05; **p < 0.01; ***p < 0.001 by paired Student's *t* test. AGR2, arginase 2; CFSE, carboxyfluorescein succinimidyl ester; COX2, cyclooxygenase 2; CSF2RB, colony stimulating factor 2 receptor subunit beta; IL, interleukin; PDL1, programmed cell death 1 ligand 1.

To further explore the role of *LTΒR* in TAM function, we first performed RNA sequencing in TAMs treated with *LTBR* siRNA (si*LTΒR*) or control siRNA (Ctrl) after confirming its knockdown efficiency (Figure S6A). The gene set enrichment analysis (GSEA) results showed that knockdown of *LTΒR* inhibited the expression of genes involved in chemokines and chemokine receptor biogenesis and T cell exhaustion, including C‐X‐C motif chemokine ligand 1 (*CXCL1*), *CXCL2*, programmed cell death 1 ligand 1 (*PDL1*), arginase 2 (*ARG2*), and cyclooxygenase 2 (*COX2*), without significant changes in the biological process of Fc gamma receptor‐mediated phagocytosis, major histocompatibility complex pathway, as well as antigen processing and presentation (Figures 3A,B and S6B). The quantitative reverse transcription polymerase chain reaction (qRT‐PCR) results showed that knockdown of *LTΒR* inhibited the mRNA level of *CXCL1*, *CXCL2*, *PDL1*, *ARG2*, *COX2*, transforming growth factor beta receptor 1 (*TGFβR1*), colony stimulating factor 2 receptor subunit beta (*CSF2RB*), mannose receptor (*MR*), *IL10*, and transforming growth factor β (*TGFβ*) (Figure S6C,D). Meanwhile, enzyme‐linked immunosorbent assay (ELISA) confirmed that knockdown of LTΒR decreased the secretion of CXCL1, CXCL2, IL10, and TGFβ from TAMs (Figure 3C). Moreover, Western blot analysis and FACS assay showed that knockdown of LTΒR inhibited the protein level of PDL1, ARG2, COX2, TGFβR1, CSF2RB, and MR in TAMs (Figure 3D–G). Conversely, activation of *LTΒR* by agonistic *LTΒR* antibodies could promote the expression of *CXCL1*, *CXCL2*, *PDL1*, *ARG2*, *COX2*, *TGFβR1*, *TGFβR2*, *CSF2RB*, *MR*, *IL10*, and *TGFβ* (Figures 3H,I and S6E,F). Thus, *LTΒR*‐mediated signaling could regulate the expression of *CXCL1*, *CXCL2*, *PDL1*, *ARG2*, *COX2*, *TGFβR1*, *CSF2RB*, *MR*, *IL10*, and *TGFβ*.

Previous studies have reported that (1) *CXCL1* and *CXCL2* are involved in the recruitment of G‐MDSC and monocytic myeloid‐derived suppressor cell (M‐MDSC), which contribute to TISM [26, 27]; (2) *PDL1*, *ARG2*, *COX2*, *TGFβR1*, and *CSF2RB* participate in T cell exhaustion and immunosuppressive behavior of TAMs [11, 28, 29]; and (3) *MR*, *IL10*, and *TGFβ* serve as M2 phenotype markers of macrophages [10, 30]. To investigate whether *LTΒR* assists TAMs to recruit G‐MDSC and M‐MDSC, they were sorted and then co‐cultured with TAMs in a transwell system, respectively. The results showed that knockdown of *LTΒR* in TAMs attenuated the recruitment of G‐MDSC, albeit no significant changes in M‐MDSC recruitment (Figures 3J and S6G,H). On the other hand, *LTΒR* activation in TAMs promoted the recruitment of G‐MDSC (Figure S6I). Moreover, the co‐culture assay of TAMs and CD8^+^ T cells showed that disruption of *LTΒR* in TAMs improved the proliferation of CD8^+^ T cells (Figures 3K and S6J). It has been reported that TAMs induce the exhaustion and anergy of CD8^+^ T cells via activating their inhibitory receptors, such as programmed cell death protein 1 (*PD1*) and T‐cell immunoglobulin and mucin domain 3 (*TIM‐3*), which reduced CD8^+^ T cell tumoricidal activity through inhibiting interferon gamma (*IFNγ*) and granzyme B (*GZMB*) [10, 31, 32]. Indeed, TAMs and CD8^+^ T cell co‐culture assays showed that activation of *LTΒR* in TAMs promoted the expression of *PD1* and *TIM‐3*, but inhibited the expression of *IFNγ* and *GZMB* in CD8^+^ T cells (Figure S6K). Disruption of *LTBR* in TAMs exerted the opposite effects on CD8^+^ T cell function (Figure S6L). These functional studies indicated that *LTΒR* ^+^ TAMs could recruit G‐MDSC and impede the proliferation and tumoricidal activity of CD8^+^ T cells. Taken together, these results demonstrated that *LTΒR* could maintain TAM‐mediated immunosuppression of CD8^+^ T Cells.

### LTΒR maintained TAM immunosuppressive features by noncanonical NF‐κB signaling and Wnt/β‐catenin signaling

**Figure 4:**
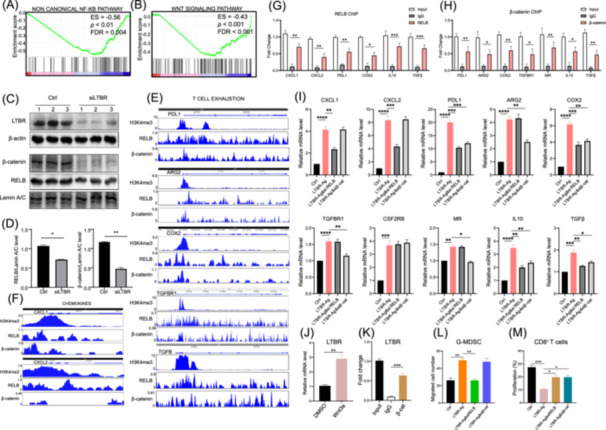
Lymphotoxin *β* receptor (LTBR) maintains tumor‐associated macrophages (TAMs) immunosuppressive behavior and M2 phenotype by noncanonical nuclear factor kappa B (NF‐κB) signalling and Wnt/β‐catenin signaling. (A, B) RNA‐sequencing data of TAMs treated with control small interfering RNA (siRNA) (Ctrl) and *LTBR* siRNA (si*LTBR*) was utilized for gene set enrichment analysis (GSEA) of noncanonical nuclear factor kappa B signaling (A) and Wnt/β‐catenin signaling (B). (C, D) After transfection of si*LTBR* or Ctrl in TAMs, the nuclear and cytoplasmic protein levels of indicated genes were detected by western blot analysis and then quantitatively compared (*n* = 3). (E, F) Chromatin immunoprecipitation (ChIP)‐seq data from the Cistrome Project were utilized to analyze potential binding sites of RELB, β‐catenin, and H3K4me3 on the promoter of genes involved in T cell exhaustion (E) and chemokines (F). (G, H) The binding of RELB (G) and β‐catenin (H) to the promoter of the indicated genes was analyzed via ChIP array (*n* = 3). (I) After activation of LTΒR by agonistic LTΒR antibodies, TAMs were transfected with *RELB* siRNA (si*RELB*), *β‐catenin* siRNA or control siRNA (Ctrl). Twenty‐four hours after transfection, the expression of indicated genes was determined by quantitative reverse transcription polymerase chain reaction (qRT‐PCR) (*n* = 3). (J) The expression of *LTBR* in TAMs treated with Wnt3a or DMSO was tested by qRT‐PCR (*n* = 3). (K) The binding of β‐catenin to the promoter of *LTBR* gene was analyzed via ChIP array (*n* = 3). (L) TAMs were treated as (I) and then co‐cultured with granulocytic myeloid‐derived suppressor cell (G‐MDSC) in a transwell system. The migration of G‐MDSC was analyzed by microscopy (*n* = 3). (M) TAMs were treated as (I) and then co‐cultured with CD8^+^ T cells. The proliferation of CD8^+^ T cells was analyzed by flow cytometry (*n* = 3). Data are shown as mean ± SEM. *p < 0.05; **p < 0.01; ***p < 0.001; ****p < 0.0001 by paired Student's *t* test (D, G, H, J, and K) or one‐way analysis of variance with Tukey's multiple comparison test (I, L, and M). AGR2, arginase 2; COX2, cyclooxygenase 2; IgG, immunoglobulin G; IL, interleukin; PDL1, programmed cell death 1 ligand 1.

Considering LTBR signaling activates both the canonical and noncanonical NF‐κB signaling pathways [33], we hypothesized that the NF‐κB signaling pathway might be a potential mechanism. GSEA results showed that *LTΒR* knockdown in TAMs affected noncanonical NF‐κB signaling rather than canonical NF‐κB signaling (Figures 4A and S7A). Notably, *LTΒR* knockdown in TAMs also disrupted Wnt/β‐catenin signaling (Figure 4B). Noncanonical NF‐κB signaling and Wnt/β‐catenin signaling regulate lung cancer progression via RELB proto‐oncogene (*RELB*) and *β‐catenin*, respectively [34, 35, 36]. Indeed, knockdown of *LTΒR* in TAMs reduced the translocation of RELB and β‐catenin into the nucleus (Figure 4C,D). Conversely, LTBR activation promoted the translocation of RELB and β‐catenin into the nucleus in TAMs (Figure S7B). In addition, chromatin immunoprecipitation (ChIP)‐seq data from the Cistrome Project (http://cistrome.org/) showed that high binding levels of *RELB* existed on the promoter of *CXCL1*, *CXCL2*, *PDL1*, *COX2*, *IL10*, and *TGFβ*, while high binding levels of *β‐catenin* existed on the promoter of *PDL1*, *ARG2*, *COX2*, *TGFβR1*, *IL10*, *MR*, and *TGFβ*, along with high binding levels of H3K4me3 (Figures 4E,F and S7C). Then, ChIP experiments further confirmed the above ChIP‐seq results (Figure 4G,H). Furthermore, knockdown of *RELB* could impede the effect of the upregulation of *CXCL1*, *CXCL2*, *PDL1*, *COX2*, *IL10*, and *TGFβ* by *LTΒR* activation. And knockdown of *β‐catenin* could attenuate the effect of the upregulation of *PDL1*, *ARG2*, *COX2*, *TGFβR1*, *IL10*, *MR*, and *TGFβ* by *LTΒR* activation (Figures 4I and S7D,E).

Moreover, we expected to address the potential upstream signaling of *LTBR* expression in TAMs. ChIP‐seq analysis showed that *β‐catenin* could bind the promoter region of *LTBR*, along with the binding peak of H3K4me3 (Figure S7F). Indeed, our results showed that activation of Wnt/β‐catenin signaling in TAMs by Wnt3a could upregulate the expression of *LTBR*, while knockdown of *β‐catenin* significantly inhibited *LTBR* expression (Figures 4J and S7G). Finally, ChIP assay confirmed that *β‐catenin* could bind the promoter region of *LTBR* (Figure 4K). Considering that *LTBR* activation promoted Wnt/β‐catenin signaling (Figures 4B,C and S7B), the above results indicated that there was a feedback regulation pathway between Wnt/β‐catenin signaling and *LTBR* expression in TAMs.

Next, we assessed whether *LTΒR* could assist TAMs to recruit G‐MDSC via noncanonical NF‐κB signaling or Wnt/β‐catenin signaling. First, TAMs were co‐cultured with G‐MDSC in a transwell system. The results showed that knockdown of *RELB* in TAMs attenuated the recruitment of G‐MDSC by *LTBR* activation, albeit no significant changes after knockdown of *β‐catenin* (Figure 4L). Moreover, the co‐culture assay of TAMs and CD8^+^ T cells showed that knockdown of *RELB* or *β‐catenin* could rescue CD8^+^ T cell proliferation after *LTBR* activation (Figure 4M). Collectively, these results indicated that *LTΒR* maintained TAM immunosuppressive activity through noncanonical NF‐κB signaling and Wnt/β‐catenin signaling.

### Knockout of *LTBR* in TAMs impedes tumor growth via disrupting TAM immunosuppressive activities and M2 phenotype

**Figure 5:**
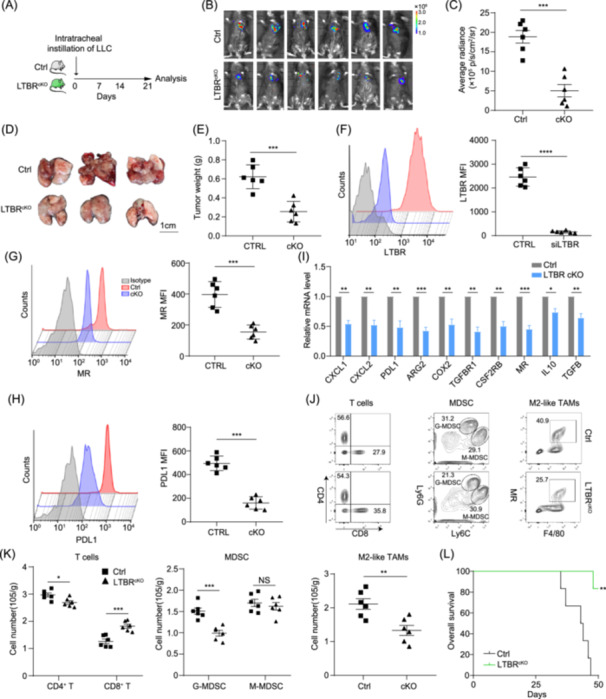
Knockout of lymphotoxin *β* receptor (*LTBR*) in tumor‐associated macrophages (TAMs) impedes tumor growth via disrupting TAM immunosuppressive activities and M2 phenotype. (A) Orthotopic lung cancer model was established by intratracheally instillation of luciferase‐carried Lewis lung carcinoma (LLC) cells in macrophage‐specific *LTBR* knockout (*LTBR* ^cKO^) mice and the control (Ctrl) mice. (B) Three weeks after LLC inoculation as (A), the growth of orthotopic lung cancer was monitored by an in vivo imaging system (*n* = 6). (C) The quantification of average radiance in each group from (B) (*n* = 6). (D) The representative images of tumors from (B) were displayed. (E) The tumor weight of mice from (D) was measured and compared (*n* = 6). (F–H) The mean fluorescence intensity of LTΒR (F), mannose receptor (G), and programmed cell death 1 ligand 1 (PDL1) (H) in TAMs isolated from (D) was measured by a fluorescence‐activated cell sorter (*n* = 6). (I) In the sorted TAMs from (D), the expression of the indicated genes was tested by quantitative reverse transcription polymerase chain reaction (*n* = 3). (J, K) The infiltration of T cells, myeloid‐derived suppressor cells (MDSC), and M2‐like TAMs was measured by flow cytometry (*n* = 6). (L) The survival curves of LLC‐bearing *LTBR* ^cKO^ and Ctrl mice were compared by log‐rank test, **p < 0.01. Data are shown as mean ± SEM. *p < 0.05; **p < 0.01; ***p < 0.001; ****p < 0.0001 using paired Student's *t* test (C, E–K). AGR2, arginase 2; COX2, cyclooxygenase 2; G‐MDSC, granulocytic myeloid‐derived suppressor cell; siLTBR, *LTBR* siRNA.

To evaluate the impact of *LTBR* ^+^ TAMs on tumor development in vivo, we used macrophage‐specific *LTBR* knockout (*LTBR* ^cKO^) mice. After confirming *LTBR* knockout efficiency, *LTBR* ^cKO^ mice and the control (Ctrl) mice were intratracheally instilled with Lewis lung carcinoma (LLC) cells to establish an orthotopic lung cancer model (Figures 5A and S8A,B). Three weeks after instillation, bioluminescence imaging showed that the tumor growth was impeded in *LTBR* ^cKO^ mice compared to Ctrl mice (Figure 5B,C). Moreover, the tumor weights of *LTBR* ^cKO^ mice significantly decreased compared with Ctrl mice (Figure 5D,E). FACS analysis validated that the expression of LTBR in TAMs from *LTBR* ^cKO^ mice was significantly inhibited compared with Ctrl mice (Figure 5F). Further analysis showed that knockout of *LTBR* in TAMs inhibited the expression of *CXCL1*, *CXCL2*, *ARG2*, *PDL1*, *MR*, *COX2*, *TGFβR1*, *CSF2RB*, *IL10*, and *TGFβ*, suggesting that disruption of *LTBR* hindered TAM immunosuppressive features and M2 phenotype (Figures 5G–I and S8A). Moreover, TIM analysis showed that higher infiltration of CD8^+^ T cells, as well as lower infiltration of M2‐like TAMs and G‐MDSC, existed in *LTBR* ^cKO^ mice compared with Ctrl mice, indicating that knockout of *LTBR* in TAMs could remodel TIM (Figure 5J,K). Meanwhile, knockout of *LTBR* in TAMs could prolong the survival of tumor‐bearing mice (Figure 5L). These results were also validated in the subcutaneous melanoma and glioma model (Figure S8C–H).

To further investigate whether *LTBR* ^+^ TAMs are important for tumor growth, Ctrl and *LTBR* ^cKO^ mice were orthotopically inoculated with LLC cells following with macrophage depletion by clodronate liposomes [37]. Three weeks after inoculation, tumor weight analysis showed that no matter clodronate liposome treatment or knockout of *LTBR* in macrophages partially hindered tumor progression, but the combination strategy showed no additive effects, indicating the critical role of *LTBR* ^+^ TAMs in LLC progression (Figure S8I–K). Altogether, our results indicated that knockout of *LTBR* in TAMs inhibited tumor growth by disrupting TAM immunosuppressive activities and M2 phenotype.

### TAM‐targeted delivery of *LTBR* siRNA disrupts TAM immunosuppressive ability and improves immunotherapy response

**Figure 6:**
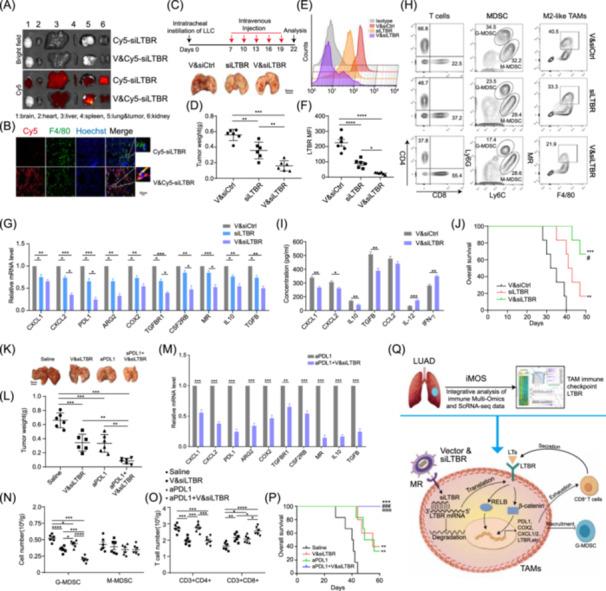
Tumor‐associated macrophage (TAM)‐targeted delivery of lymphotoxin *β* receptor (*LTBR*) small interfering RNA disrupts TAM immunosuppressive ability and improves immunotherapy response. (A) The distribution of Cy5‐labled *LTBR* siRNA (si*LTBR*) in different organs of tumor‐bearing mice was observed by IVIS Lumina system at 6 h after tail vein administration of naked Cy5‐si*LTBR*, and vector (V)＆Cy5‐si*LTBR*. (B) Tumor sections from tumor‐bearing mice described in (A) were stained with fluorescein isothiocyanate (FITC)‐F4/80 antibody and Hoechst, followed by images acquisition with confocal microscopy. (C, D) The orthotopic lung cancer model was established by intratracheally instillation of Lewis lung carcinoma (LLC) cells. One week after instillation, tumor‐bearing mice were intravenously injected with V&siCtrl, si*LTBR*, and V&si*LTBR* every 3 days for five times. Three days after the last treatment, the tumors were collected (*n* = 6). The representative images and quantitative comparison of tumor weights are shown in (C) and (D), respectively. (E, F) The mean fluorescence intensity of LTΒR in TAMs from mice treated as (C) was measured and compared by a fluorescence‐activated cell sorter (FACS) (*n* = 6). (G) TAMs from mice treated as (C) were sorted. The expression of the indicated genes in these sorted TAMs was measured by quantitative reverse transcription polymerase chain reaction (qRT‐PCR) (*n* = 3). (H) The infiltration of T cells, myeloid‐derived suppressor cells (MDSC), and M2‐like TAMs was measured by FACS. (I) Serum from tumor‐bearing mice treated as (C) was collected, and the concentration of the indicated cytokines was determined by enzyme‐linked immunosorbent assays (*n* = 3). (J) After V&siCtrl, si*LTBR*, or V&si*LTBR* treatment, the survival curves of tumor‐bearing mice were analyzed by log‐rank test. Versus V&siCtrl: **p < 0.01; ***p < 0.001; versus si*LTBR*: ^#^ *p* < 0.05 by log‐rank test. (K) After the establishment of the orthotopic lung cancer model, tumor‐bearing mice were treated with saline, V&si*LTBR*, programmed cell death 1 ligand 1 (*PDL1*) antibody (a*PDL1*), and V&si*LTBR* + a*PDL1* every 3 days for five times. After that, the tumors were dissected and photographed. (L) The tumor weight of different treatment as (K) were compared (*n* = 6). (M) In sorted TAMs from (K), the expression of genes involved in chemotaxis, T cell exhaustion, and immune suppression was measured by qRT‐PCR (*n* = 3). (N, O) After different treatments as (K), the proportion of MDSC (N) and T cells (O) was measured by FACS (*n* = 6). (P) The survival curves of tumor‐bearing mice were observed after treatment with different drugs as shown in (K); versus saline: **p < 0.01; ***p < 0.001; versus V&si*LTBR*: ^###^ *p* < 0.001; versus a*PDL1*: p@@@ < 0.001 by log‐rank test. (Q) The schematic diagram shows that integrative analysis of immune multiomics data and single cell RNA‐seq data (iMOS) reveals *LTΒR* as the immune checkpoint of TAMs to exhaust cytotoxic CD8^+^ T cells and recruit G‐MDSC by noncanonical nuclear factor kappa B signaling and Wnt/β‐catenin signaling. Data are shown as mean ± SEM. *p < 0.05; **p < 0.01; ***p < 0.001, ****p < 0.0001 by paired Student's *t* test (I and M) or one‐way analysis of variance with Tukey's multiple comparison test (D, F, G, L, N, and O).

Then, we wondered whether TAM‐targeted inhibition of *LTΒR* could affect tumor growth and immunotherapy response. Due to *MR* specifically expressed in TAMs (Figure S9A), we utilized a TAM‐targeted siRNA delivery system (vector) with high affinity for *MR* as previously reported [38] (Figure S9B). The orthotopic lung cancer model was established and then intravenously injected with Cy5‐labeled si*LTBR* (Cy5‐si*LTBR*) and vector‐loaded Cy5‐labeled si*LTBR* (V&Cy5‐si*LTBR*). After that, in vivo live imaging showed that V&Cy5‐si*LTBR* was enriched mostly in lung cancer tissue rather than in other tissues (Figure 6A). Immunofluorescence assay indicated that this system could deliver si*LTBR* specifically into TAMs (Figure 6B). Then, the orthotopic lung cancer model was established by intratracheally instillation of LLC cells. One week after instillation, tumor‐bearing mice were intravenously injected with V&siCtrl, si*LTBR*, and V&si*LTBR* every 3 days for five times. After five treatments, the tumor weight of mice administrated with V&si*LTBR* decreased much more than that of mice administrated with only si*LTBR* or V&siCtrl (Figure 6C,D). Moreover, FACS assay showed that V&si*LTBR* treatment had a stronger capacity to knockdown the LTΒR expression in TAMs than the systematic delivery of si*LTBR* (Figure 6E,F). In sorted TAMs, we found that TAM‐targeted delivery of *LTBR* siRNA could significantly inhibit the expression of *CXCL1*, *CXCL2*, and genes involved in T cell exhaustion and immune suppression, such as *PDL1*, *IL10*, and *TGFβ* (Figures 6G and S9C). Further TIM analysis showed that V&si*LTBR* treatment improved the infiltration of CD8^+^ T cells and reduced the infiltration of M2‐like TAMs and G‐MDSC, compared with the only si*LTBR* or V&siCtrl treatment (Figures 6H and S9D–F). Mouse serum cytokine arrays further demonstrated that V&si*LTBR* treatment decreased the concentration of *CXCL1*, *CXCL2*, *IL10*, and TGF*β* and increased the concentration of *IL12* and *IFNγ* compared with V&siCtrl treatment (Figure 6I). And the mice treated with V&si*LTBR* had a longer survival time than those treated with only si*LTBR* or V&siCtrl (Figure 6J). These results indicated TAM‐targeted delivery of si*LTBR* could abrogate TAM immunosuppressive capacity and remodel the proportion of tumor infiltrating immune cells by increasing CD8^+^ T cells as well as decreasing M2‐like TAMs and G‐MDSC, eventually resulting in tumor impedance.

Due to *LTBR* ^+^ TAMs involved in immunotherapy failure, we wondered whether TAM‐targeted inhibition of *LTΒR* could affect the therapeutic effect of ICI. First, the orthotopic lung cancer model was established and then intravenously injected with saline, V&si*LTBR*, *PDL1* monoclonal antibody (a*PDL1*), and V&si*LTBR* plus a*PDL1* (Figure S10A). After five treatments, the tumor weight of V&si*LTBR* plus a*PDL1*‐treated mice was significantly lower than that of only V&si*LTBR* or a*PDL1‐*treated mice, suggesting that TAM‐targeted delivery of si*LTBR* could enhance the therapeutic effect of *PDL1* antibody (Figure 6K,L). Moreover, in sorted TAMs, we found that the expression of *CXCL1*, *CXCL2*, *PDL1*, *ARG2*, *COX2*, *TGFβR1*, *CSF2RB*, *MR*, *IL10*, and *TGFβ* in V&si*LTBR* plus a*PDL1* treated group was significantly lower than that in only the a*PDL1*‐treated group (Figure 6M). Compared with only V&si*LTBR* or a*PDL1*‐treated group, higher infiltration of CD8^+^ T cells, as well as lower infiltration of M2‐like TAMs and G‐MDSC, existed in V&si*LTBR* plus a*PDL1*‐treated group (Figures 6N,O and S10B,C). Last but not least, V&si*LTBR* plus a*PDL1*‐treated mice had a longer survival time than only V&si*LTBR* or a*PDL1*‐treated mice (Figure 6P). These results suggested that inhibition of *LTΒR* in TAMs could improve the therapeutic effect of ICI via blocking TAM immunosuppressive activity.

## DISCUSSION

To identify and select appropriate immune checkpoints for most cancer patients, including LUAD, remains a great challenge although ICI‐based precision immunotherapy has been applied efficiently in some tumors. Recently, multiomics and scRNA‐seq technologies pave the way to find more tumor biomarkers and targets. However, several multiomics investigations of LUAD only focused on the mutation, rearrangement, and epigenetic modification of driver genes including *EGFR*, *KRAS*, and *ALK* [8, 39], but little attention has been paid to TIM. Moreover, the scRNA‐seq method brings unique insights into TIM [40], and the comprehensive interpretation of multiomics and scRNA‐seq data will be a promising option. In this study, we develop a unique immune checkpoint discovery pipeline iMOS and successfully screen out *LTΒR* as a novel immune checkpoint on TAMs (Figure 1). Recent evidence shows that *LTBR* is mostly expressed on myeloid cells by analyzing melanoma scRNA‐seq data [41]. Similarly, we find that *LTBR* is dominantly expressed in myeloid cells of LUAD, especially high expression in TAMs (Figure 1). Moreover, the infiltration of *LTBR* ^+^ TAMs is associated with LUAD stages, immunotherapy failure, and poor prognosis (Figure 2). More importantly, disruption of *LTΒR* in TAMs hinders tumor growth and even enhance the efficacy of immunotherapy through reversing TAM‐mediated immunosuppression, for instance, boosting CD8^+^ T cells and repressing G‐MDSC as well as M2‐like TAMs (Figure 5). These results show that iMOS could be a powerful method to discover more alternative immune checkpoint molecules, which can be applied to other cancers.

To our knowledge, the role of *LTBR* in LUAD progression, especially in TAMs, has not been reported. In this study, we find that the infiltration of *LTBR* ^+^ TAMs is associated with LUAD stages and poor prognosis. Importantly, the number of *LTBR* ^+^ TAMs in immunotherapy nonresponders is also higher than that in responders, indicating that *LTBR* ^+^ TAMs participate in LUAD immunotherapy resistance (Figure 2). Previous studies have reported that *LTBR* is involved in macrophage differentiation and function [17, 18]. In lymph node and spleen, the differentiation of CD169^+^ macrophages critically depend on *LTBR* signaling [17]. Considering LTα1β2, the ligand of *LTBR*, are mainly expressed on lymphoid cells, such as T, B, and NK cells (Figure S4B), we speculate that the interaction of LTα1β2 and *LTBR* might mediate an essential communication between lymphocytes and macrophages in LUAD. In an inflammatory study, Wimmer et al. found that T cell‐derived LTα1β2 repress the pro‐inflammatory activity of macrophages via binding to *LTBR* [18], implying that *LTBR* activation can serve as a negative feedback signal to enhance macrophage‐mediated immunosuppression of T cells. In this study, we firstly demonstrate that *LTBR* activation can enhance TAM‐mediated immunosuppression of CD8^+^ T cells via upregulating immunosuppressive molecules, including *PDL1*, *ARG2*, *COX2*, *IL10*, and *TGFβ* (Figures 3H,I and S6E). Notably, *PDL1* expressed by TAMs can directly induce the exhaustion of CD8^+^ T cells [30, 31]. *ARG2* expressing TAMs compete with T cells for arginine and thus disrupt CD8^+^ T cell metabolism and inhibit their proliferation [42]. Moreover, *COX2* enhances TAM‐mediated T cell exhaustion via producing prostaglandin E2 (PGE2) [43]. It is reported that *IL10* and *TGFβ* secreted by TAMs can directly inhibit the cytotoxicity of CD8^+^ T cells [44, 45]. The above evidence further supports that *LTBR*‐activated TAMs induce the exhaustion of CD8^+^ T cells via upregulating these immunosuppressive molecules. Indeed, this role of *LTΒR* in TAMs functions still needs to be validated in other tumor models.

Accumulating evidence shows that noncanonical NF‐κB signaling is involved in tumor initiation, development, and metastasis [46, 47, 48]. Meanwhile, the activation of noncanonical NF‐κB signaling predicts poor survival and resistance to therapy [49, 50]. However, the involvement of noncanonical NF‐κB signaling in TAMs function is less well documented. Herein, we find that *RELB*, the key transcription factor of noncanonical NF‐κB signaling, can bind to the promoter region of chemokines (*CXCL1* and *CXCL2*), T cell exhaustion‐related genes (*PDL1* and *COX2*), and M2 phenotype genes (*IL10* and *TGFβ*). Knockdown of *RELB* in TAMs can attenuate the expression of genes mentioned above after *LTΒR* activation. Unexpectedly, we also find that inhibition of *LTΒR* could downregulate Wnt/β‐catenin signaling in TAMs (Figure 4). Previous studies including ours have demonstrated that activated Wnt/β‐catenin signaling contributes to macrophage M2 phenotype and exerts pro‐tumor activities [36, 51, 52]. Herein, knockdown of *β‐catenin* in TAMs partly reverses the upregulation of T‐cell exhaustion‐related genes (*PDL1*, *ARG2*, *TGFβR1*, and *COX2*) and M2 phenotype genes (*MR*, *IL10*, and *TGFβ*) after *LTΒR* activation (Figure 4I), indicating that *LTΒR* activation may enhance TAM immunosuppressive activity and M2 phenotype partly via Wnt/β‐catenin signaling. However, whether *LTΒR* activation affects Wnt/β‐catenin signaling directly or indirectly needs to be revealed in the future.

Interestingly, we find that Wnt signaling can be one of the upstream signaling pathway to regulate *LTBR* expression via *β‐catenin* binding to the promoter of *LTBR* (Figure 4J,K). Wnt/β‐catenin signaling plays a significant role in lung tumor initiation and progression [35]. Meanwhile, Wnt/β‐catenin signaling also mediates the crosstalk between tumor cells and TAMs [53]. Recent evidence shows that Wnt/β‐catenin signaling hinders antitumor immune responses and leads to immunotherapy resistance [54]. However, Wnt signaling inhibitors also bring systematic side effects, including neurological complications, kidney injury, and intestinal toxicity, due to the essential role of Wnt signaling in the tissue development and homeostasis [54]. Thus, the targeted intervention strategy of Wnt signaling needs to be developed in the future studies. Indeed, TAM‐targeted intervention system in this work might be an alternative choice.

Although ICIs have been applied to tumor therapy, some patients show no response after ICI treatment due to the dominant myelosuppressive microenvironment, especially a large number of TAMs [29, 41]. Several TAM‐targeting agents, such as anti‐*CSF1R* antibodies, *CCR2* inhibitors, anti‐*CD40* agonists, and *CD47/SIRPα* blockers, have been used in clinical trials due to their roles in blocking macrophage survival and recruitment, abolishing immunosuppression, and enhancing phagocytosis, but some drawbacks should be paid more attentions to [11, 28, 30, 55]. For example, interruption of *CSF‐1R* can cause the accumulation of monocyte‐derived macrophages leading to tumor recurrence [56]. Owing to highly expressed *CD47* on normal red blood cells, disruption of *CD47/SIRPα* axis may result in lethal autoimmune hemolytic anemia [57, 58]. Thus, it is urgent to develop more precise and specific TAM‐targeted strategies for cancer immunotherapy. In this study, using iMOS, we identify *LTΒR* as a novel immune checkpoint on TAMs. Notably, the infiltration of *LTΒR* ^+^ TAMs is associated with LUAD stages and poor prognosis. Moreover, in LUAD patients treated with ICIs, the expression of *LTΒR*, as well as the infiltration of *LTΒR* ^+^ TAMs, in nonresponders is much higher than that in responders. Meanwhile, ICI‐treated patients with higher *LTΒR* expression possess worse prognosis than those with lower *LTΒR* expression (Figure 2). These results suggest that *LTBR* can serve as not only an immune checkpoint on TAMs but also a potential biomarker for the prediction of immunotherapy responses and clinical outcomes. More importantly, TAM‐targeted delivery of *LTΒR* siRNA improves the efficacy of ICI via reversing TAM‐mediated immunosuppression, like boosting CD8^+^ T cells and inhibiting G‐MDSC (Figure 6). Thus, our study also brings out a potential combinational therapy strategy with ICIs for LUAD treatment.

We acknowledge several limitations of our study. First, bone marrow‐derived macrophages (BMDMs) are co‐cultured with lung cancer cells and then used as alternative TAMs due to few primary cells isolated from relatively small cancer tissues. Although some teams have applied the same method to mimic TAMs for further functional and mechanistic investigations [45, 50], to obtain a large number of TAMs is worth of being explored, for example, to adopt tumor organoids consisting of multiple cell types. Second, the precise mechanism of Wnt/β‐catenin signaling activated by *LTΒR* needs further investigations. Finally, we could not exclude the role of *LTBR* in other immune cells, like dendritic cells. Moreover, it is better to use macrophage‐specific conditional *LTBR* knockout mice to uncover the role of *LTΒR* ^+^ TAMs in other tumor models.

## CONCLUSION

This study sets up an immune checkpoint discovery pipeline iMOS and successfully screens out *LTΒR* as a novel TAM immune checkpoint; the mechanistic investigations unveil that *LTΒR* maintains TAM immunosuppressive activity and M2 phenotype by noncanonical NF‐κB and Wnt/β‐catenin signaling pathways; preclinical studies demonstrate that the infiltration of *LTΒR* ^+^ TAMs is associated with LUAD stages, immunotherapy resistance, and poor prognosis, and targeting *LTBR* ^+^ TAMs can be a promising strategy for improving immunotherapy.

## METHODS

### Human studies

The LUAD tissue microarray was purchased from Wuhan Servicebio. The clinical LUAD paraffin‐embedded tissues in TD‐FOREKNOW phase 2 multicenter RCT [3] (ClinicalTrials.gov: NCT04338620) were obtained from the Department of Thoracic Surgery, Tangdu Hospital, Fourth Military Medical University. The study was approved by the Ethics Committee of Tangdu Hospital of Fourth Military Medical University and was conducted according with the Declaration of Helsinki. Informed consent was obtained from all the involved patients. The clinical data of the patients are summarized in Table S1.

### Animal studies

C57BL/6 mice were maintained in a specific pathogen‐free facility. The animals were housed at 22 ± 2°C, humidity of 55 ± 10%, and 12/12 h light/dark cycle with free access to normal chow food and water. To generate macrophage‐specific Cas9‐expressed mice, Rosa26‐floxed STOP‐Cas9 knockin mice (stock #026175; Jackson Laboratories) were bred with LyzM‐Cre mice (stock #019096; Jackson Laboratory). To generate macrophage‐specific knockout of *LTBR*, macrophage‐specific Cas9‐expressed mice were intravenously injected with lentivirus carrying single guide RNA for *LTBR* (1 × 10^8^ particles per mouse). Macrophage‐specific Cas9 transgenic mice injected with lentivirus carrying empty vectors were utilized as controls. At the beginning of the experiments, animals weighed 24 ± 2 g. All the animal experiments were approved by the Animal Experiment Administration Committee of the Fourth Military Medical University and carried out in accordance with the Guide for the Care and Use of Laboratory Animals prepared by the National Academy of Sciences that is published by the National Institutes of Health to ensure the ethical and humane care of the animals.

LLC cells were purchased from the American Type Culture Collection. For the orthotopic lung cancer model, intratracheal instillation of 1 × 10^6^ LLC and quantification were performed as previously described [52]. Lung cancer growth was measured by using an IVIS imaging system (Xenogen, Perkin‐Elmer). For the subcutaneous melanoma mouse model, 2 × 10^6^ B16 cells were injected subcutaneously on the rear back of C57BL/6 mice. Tumor volume was monitored by measuring the long (*L*) and short (*S*) tumor diameters with a sliding caliper (tumor size = 0.513 × *L* × *S* ^2^). The mice were euthanized at the indicated time via intraperitoneal injection of pentobarbital sodium (60 mg/kg), and then, the weight of tumors was measured.

To deplete macrophage in vivo, mice were intravenously injected with 1 mg clodronate or control liposomes per 20 g body weight every 4 days for five times, and flow cytometry was used to validate the efficiency.

### Eukaryotic cells studies

BMDM, RAW264.7 cells, and in vitro cultured TAMs were obtained and cultured as previously described [36, 38, 52]. Briefly, mouse femur and tibia bones were flushed with Dulbecco's modified eagle medium (DMEM) (Gibco), and red blood cells were lysed using red blood cell lysis buffer (Beyotime). After counting, 20 million bone marrow (BM) cells were seeded per 15 cm nontissue culture plates in DMEM with 10% fetal bovine serum (FBS) (Gibco), 2 mmol/L l‐glutamine (Gibco), 25 ng/mL murine macrophage‐colony‐stimulating factor (SinoBioa), and 100 U/mL penicillin/streptomycin (Gibco). After 3 days of differentiation, nonadherent cells were washed off with room temperature DMEM and then cultured for another 3 days to obtain a homogeneous population of BMDM, as determined by FACS.

For obtaining in vitro cultured TAMs, we first harvested LLC with Trypsin‐EDTA solution (Beyotime), washed them once with cell medium (DMEM supplemented with 10% FBS and 100 U/mL penicillin/streptomycin), and subsequently resuspended them in BMDM medium. Then, we co‐cultured BMDM and LLC in a 1:1 ratio (1 × 10^5^ BMDM:1 × 10^5^ LLC per six‐well plate in 2 mL of medium) in BMDM medium for 3 days. Following that, the cell medium in the culture dish was removed, and the remaining cancer cells were detached by using 500 mL of Trypsin‐EDTA solution (Beyotime) for 3 min, which were then discarded from the culture dish. Subsequently, we washed the macrophages in the six wells three times and replenished macrophage medium. After coculturing with LLC, TAMs were obtained for further investigations, including FACS, qRT‐PCR, western blot analysis, and so on.

MDSC, M‐MDSC (CD11b^+^Ly6C^hi^Ly6G^‐^), and G‐MDSC (CD11b^+^Ly6C^low^Ly6G^+^) were obtaining as previously reported [59]. Briefly, the BM cells were induced with GM‐CSF (25 ng/mL) and IL‐6 (25 ng/mL) (SinoBio) for 4 days to obtain MDSC, which were confirmed by FACS. M‐MDSC (CD11b^+^Ly6C^hi^Ly6G^‐^) and G‐MDSC (CD11b^+^Ly6C^lo^Ly6G^+^) were sorted from MDSC by FACS AriaIII flow cytometer (BD Immunocytometry Systems) and then seeded on the transwell chamber for further migration assays.

### RNA extraction and qRT‐PCR analysis

The total RNA was extracted from the cells using TRIzol reagent (Invitrogen) according to the manufacturer's instructions. Complementary DNA (cDNA) library was obtained by using the HiScriptII Q RT SuperMix Reagent Kit (Vazyme). qRT‐PCR assays were performed with the ChamQ SYBR qPCR Master Mix Kit (Vazyme) using an ABI PRISM 7500 Real‐time PCR system (Life Technologies), and β‐actin was used as the internal control. The qRT‐PCR primers are shown in Supporting Information.

### Western blot analysis

Whole cell lysates were harvested by using RIPA buffer containing a protease inhibitor cocktail (Beyotime). Then, nucleic and cytoplasmic proteins were extracted using the extraction kit (Beyotime) according to the manufacturer's instructions, respectively. Protein concentrations were determined with BCA Protein Assay kit (Pierce). Samples were separated by sodium dodecyl‐sulfate polyacrylamide gel electrophoresis and blotted on polyvinylidenefluoride membranes. Membranes were blocked with 5% skim milk solution for 1 h and then probed with primary antibodies and secondary antibodies, as listed in Supporting Information. Protein blots were developed by using ChemiScope instrument (Clinx Science Instruments Co. Ltd.).

### Immunofluorescence

The fixed tumor tissues of the mice and clinical specimens were blocked with 5% BSA after antigen retrieval. The sections were incubated with different antibodies and stained with Hoechst 33342. The sections were photographed using a laser scanning confocal microscope (FV‐1000; Olympus). The antibodies used for immunofluorescence are listed in Supporting Information.

### Migration assays

TAMs were seeded into the 12‐well plate and transfected with si*LTBR* or the Ctrl. Meanwhile, 2 × 10^4^ M‐MDSC (CD11b^+^Ly6C^hi^Ly6G^−^) or G‐MDSC (CD11b^+^Ly6C^lo^Ly6G^+^) were suspended in 200 μL serum‐free DMEM and seeded on the upper transwell chamber. After incubating at 37°C for 12 h, the noninvading cells were scrubbed away by a cotton swab. Then, the cells penetrating through the transwell chamber were labeled with crystal violet (Beyotime) and counted with a microscope.

### T cell proliferation

The spleens and lymph nodes of mice were grinded and filtered with 70 mm nylon membrane to obtain single‐cell suspension. After red blood cell lysis, CD8^+^ T cells (7AAD^−^CD3^+^CD4^−^CD8^+^) were sorted by FACS AriaIII flow cytometer (BD Immunocytometry Systems). The sorted CD8^+^ T cells (5 × 10^4^) were labeled with carboxyfluorescein succinimidyl ester (CFSE) (5 nM) and cultured in 96‐well plates coated with CD3 and CD28 antibodies (Biolegend) to activate these T cells. Then, TAMs (1 × 10^5^), transfected with si*LTBR* or the control, were co‐cultured with activated CD8^+^ T cells for 3 days. T cell proliferation was detected by FACS and evaluated by the reduction in the CFSE fluorescence intensity.

### Flow cytometry

Cells were stained with different antibodies as listed in Supporting Information. FACS analysis was performed with routine protocols using a FACS CantoPlus or FACS AriaIII flow cytometer (BD Immunocytometry Systems). The data were analyzed with FlowJo vX.0.6 software (FlowJo, LLC). The dead cells were excluded by 7‐AAD staining.

### ELISA

The amount of CXCL1, CXCL2, CCL2, IL‐12, TGFβ, IL‐10, and IFNγ in the serum with different treatments was measured using the ELISA kit (Invitrogen) according to the supplier's protocol. The ELISA kit has been listed in Supporting Information.

### TCGA data analysis

LUAD multiomics data sets (including somatic mutation, copy number, DNA methylation, transcriptional expression profile) and relevant clinical information were downloaded from the TCGA data portal (https://tcga-data.nci.nih.gov/tcga/) in December 2018. Immune scores and stromal scores were calculated by applying the ESTIMATE algorithm [19] to the downloaded data sets. The expression of *LTBR*, *LTα*, *LTβ*, and *LIGHT* in different LUAD tissues was analyzed and tested by unpaired student's *t* test or one‐way analysis of variance (ANOVA) with Tukey's multiple comparison test. The OS from the TCGA Pan‐Cancer Clinical Data Resource [60] were utilized to investigate LUAD patients' clinical outcomes. Survival curves were tested by the Kaplan–Meier method, and statistical significance was determined by the log‐rank (Mantel‐Cox) test. *p* < 0.05 was considered as statistical significance. Four independent data sets of LUAD expression data (GSE8894 [20], GSE13213 [21], GSE43767 [22], and GSE68465 [23]) as well as their relevant clinical information (Table S1) were downloaded from Gene Expression Omnibus for further validation.

### SNV analysis

SNV analysis was performed by using gene set cancer analysis (GSCA) [61] website (http://bioinfo.life.hust.edu.cn/GSCA/#/) according to their instructions. Briefly, the SNV percentage was calculated by the number of mutated sample/number of cancer sample. SNV waterfall plot was generated by maftools. R package survival was used to estimate survival difference between mutated and unmutated genes. Log rank test was also performed, and *p* < 0.05 was considered as significant.

### CNV analysis

CNV analysis was performed by using GSCA [61] website (http://bioinfo.life.hust.edu.cn/GSCA/#/) according to their instructions. Briefly, the mRNA expression and CNV data were merged by TCGA barcode. The association between paired mRNA expression and CNV was tested based on Person's correlation coefficient, and *p* value was adjusted by FDR. The survival analysis of CNV changes was tested by the log‐rank test.

### Methylation analysis

The survival analysis of the indicated gene methylation in LUAD was performed by using MethSurv website (https://biit.cs.ut.ee/methsurv/) according to their instructions.

### Analysis of DEGs

DEGs between high and low immune scores were screened out using R package limma (FC > 1.5 and *p* < 0.05). Clustering was performed using Gene Cluster V3.0. Heatmaps were drawn by using ComplexHeatmap package [62].

### Pan‐cancer survival analysis

The pan‐cancer survival analysis of the indicated genes was performed using TISIDB website (http://cis.hku.hk/TISIDB) according to their instructions.

### GO and KEGG analysis

GO and KEGG analysis of the indicated genes were performed using Metascape website (http://metascape.org) according to their instructions.

### scRNA‐seq data processing and analyzing

The lung cancer scRNA‐seq data from GEO database (GSE131907) and normal lung scRNA‐seq data (GSE134355) were processed using the Cell Ranger Single‐Cell Software Suite against the GRCh38 human reference genome. The processed data generated from Cell Ranger were utilized for further analysis. Then, quality control was applied to cells according to three metrics, including the total unique molecular identifiers (UMI) count, number of detected genes, and proportion of mitochondrial gene count per cell. Subsequently, cells with below 2000 UMI count and 500 genes or above 10% mitochondrial gene count were filtered out. To wipe off potential doublets, cells with more than 40,000 UMI count and 5000 detected genes were also filtered out. After that, we applied the library‐sized correction method to normalize the raw data with normalize_total function in Scanpy [63]. Then, the logarithmized normalized count data were utilized for downstream analysis.

Normalized scRNA‐seq data were processed for dimension reduction and unsupervised clustering with Scanpy [63]. First, 2000 highly variable genes were selected for downstream analysis utilizing scanpy.pp.highly_variable_gene function with parameter “n_top_genes=2000.” Second, effects of the total count per cell, the percentage of mitochondrial gene count, and the percentage of count for heat shock protein‐associated genes were regressed out with scanpy.pp.regress_out function. Finally, a principal component analysis matrix with 100 components was utilized to reveal the main axes of variation and denoise the data utilizing scanpy.tl.pca function with parameter “svd_solver=‘arpack’, n_comps=100.”

To visualize the results, the dimensionality of the processed scRNA‐seq data was reduced by utilizing Uniform Manifold Approximation and Projection implemented in scanpy.tl.umap function with the default parameters. For clustering single cells, we utilized an unsupervised graph‐based clustering algorithm called Leiden. The cluster‐specific marker genes were identified by using the scanpy.tl.rank_genes_groups function with default parameters [64].

### Spatial transcriptomics analysis

The spatial transcriptomics data of lung cancer were downloaded from databases CROST (https://ngdc.cncb.ac.cn/crost/home), A Comprehensive Repository of Spatial Transcriptomics [65]. Quality control was performed on the raw data to take away low‐quality reads. The Spliced Transcripts Alignment was utilized to align the sequencing reads to the reference genome [66]. Then, the transcriptome positions of cells were matched with spatial coordinates. Dimensionality reduction, clustering, spatially variable gene analysis, cell type annotation analysis, spatial correlation, and colocalization analysis were performed as previously reported [67].

### Bulk RNA sequencing

Total RNA of TAMs treated by siCtrl and si*LTBR* was extracted with TRIzol reagent (Invitrogen) following the manufacturer's instructions. Poly (A) RNA was purified from 1 mg total RNA using Dynabeads Oligo (dT) 25–61005 (ThermoFisher). After that, the poly(A) RNA was fragmented into small pieces using Magnesium RNA Fragmentation Module (NEB) under 94°C 5–7 min. Subsequently, the cleaved RNA fragments were reverse transcribed to synthesize the cDNA with SuperScript™ II Reverse Transcriptase (cat. 1896649; Invitrogen), which were next used to synthesize U‐labeled second‐stranded DNAs with *Escherichia coli* DNA polymerase I (NEB), RNase H (NEB), and deoxyuridine triphosphate solution (Thermo Fisher). After the heat‐labile UDG enzyme (NEB) treatment of the U‐labeled second‐stranded DNAs, the ligated products are amplified with PCR by the following conditions: initial denaturation at 95°C for 3 min; eight cycles of denaturation at 98°C for 15 s, annealing at 60°C for 15 s, and extension at 72°C for 30 s; and then final extension at 72°C for 5 min. At last, we performed the 2 × 150 bp paired‐end sequencing (PE150) on an illumina Novaseq™ 6000 (LC‐Bio Technology Co. Ltd.).

### Bioinformatics analysis of RNA‐seq data

Fastp software was used to remove the reads that contained adaptor contamination, low‐quality bases, and undetermined bases with a default parameter. We used HISAT2 to map reads to the reference genome of *Mus musculus* GRCm39. The mapped reads of each sample were assembled using StringTie with default parameters. Then, all transcriptomes from all samples were merged to reconstruct a comprehensive transcriptome using gffcompare package. After the final transcriptome was generated, StringTie and was used to estimate the expression levels of all transcripts. StringTie was used to perform expression level for mRNAs by calculating fragments per kilobase million (FPKM) (FPKM = [total_exon_fragments/mapped_reads (millions) × exon_length(kB)]). The DEGs were selected with FC > 2 or FC < 0.5 and with parametric *F* test comparing nested linear models (*p* < 0.05) by R package edgeR (https://bioconductor.org/packages/release/bioc/html/edgeR.html).

### GSEA

GSEA was performed using GSEA software 53 (version 4.3.2) in conjunction with the Molecular Signature Database (version 7.4). One thousand permutations were used.

### Immune cell infiltration analysis

The correlation between immune cell infiltration and *LTBR* expression level in LUAD was analyzed using TIMER2.0 website according to their instructions.

### ChIP

The Chip‐seq analysis of H3K4me3, *RELB*, and *β‐catenin* was performed by using Cistrome database [68] (http://cistrome.org) according to their instructions. ChIP experiments were performed as previously reported [38, 69].

### TAM‐targeted nucleic acid drug delivery system

Cationic konjac polysaccharide (cKGM) and PEG‐His‐modified alginate (PHA) were produced as previously reported [70, 71]. The *LTBR* siRNA and their control were purchased from RiboBio Biotech. cKGM and *LTBR* siRNA complex was formed by mixing 5 mg/mL of siRNA solution with 5 mg/mL of cKGM solution at the ratio of 1:3. The cKGM and siRNA complex solution and 5 mg/mL PHA saline solution was mixed at the ratio of 1:1 to form the triple complex (Vector and siRNA). Immunofluorescence staining was applied to determining the endocytosis of cKGM and siRNA complex, and the biodistribution of the nucleic acid drug was tested as previously reported [71]. Tumor‐bearing mice were intravenously administrated with vector and siRNA at a dose of 2 μg siRNA/g body weight every 3 days from day 7 after the injection of cancer cells. The tumor tissues were harvested for further analysis.

### Statistical analysis

The data were analyzed with GraphPad Prism 8.0.1 software. The unpaired student's *t* test, paired *t* test, or one‐way ANOVA with Turkey's multiple comparison test were performed for the comparisons among groups. Survival curves were tested by the Kaplan–Meier method, and statistical significance was determined by the log‐rank (Mantel‐Cox) test. Pearson correlation coefficients and *p* values were computed with the rcorr function in Hmisc R package. *p* < 0.05 was considered statistically significant (*p < 0.05, **p < 0.01, ***p < 0.001, ****p < 0.0001 in figures).

## AUTHOR CONTRIBUTIONS

Hongyan Qin, Liang Wang, and Yan Chen designed the overall experiments and had unrestricted access to all data and wrote the manuscript, with support from all the co‐authors. Liang Wang, Jieyi Fan, Sifan Wu, and Shilin Cheng performed the experiments. Junlong Zhao, Chunchen Gao, Yiyang Hu, Fan Fan, Qiqi Sheng, Pengjun Liu, and Sifan Wu performed statistical analyses. Liang Wang, Jieyi Fan, Sifan Wu, Zhe Jiao, Yong Zhang, Jie Lei, and Tiaoxia Wei collected biological samples and recorded the immunotherapy responses and clinical outcomes of the patient over the years. All authors have read the final manuscript and approved it for publication.

## CONFLICT OF INTEREST STATEMENT

The authors declare no competing interests.

## ETHICS STATEMENT

This study was approved by the Ethics Committee of Tangdu Hospital of Fourth Military Medical University (No. 2020003‐153) and was conducted according with the Declaration of Helsinki. Informed consent was obtained from all the involved patients.

## Supporting information


**Figure S1.** Immune scores are utilized to screen out immune‐related genes in LUAD.
**Figure S2.** The immune‐related prognostic genes were screened in TCGA and GEO database.
**Figure S3.** iMOS analysis reveals *LTBR* as a potential immune‐related mediator in LUAD.
**Figure S4.** iMOS analysis reveals *LTBR* as a potential TAMs immune checkpoint in LUAD.
**Figure S5.** *LTB*R is associated with LUAD stages, clinical prognosis and immunotherapy failure.
**Figure S6.** *LTBR* maintains TAMs immunosuppressive activity and M2 phenotype.
**Figure S7.** *LTBR* maintains TAM immunosuppressive behavior and M2 phenotype by noncanonical NF‐κB signalling and Wnt/β‐catenin signaling.
**Figure S8.** Knockout of *LTBR* in TAMs impedes tumor growth via disrupting TAM immunosuppressive activities and M2 phenotype.
**Figure S9.** TAM‐targeted delivery of *LTBR* siRNA disrupts TAM immunosuppressive ability and improves immunotherapy response.
**Figure S10.** TAM‐targeted delivery of *LTBR* siRNA disrupts TAM immunosuppressive ability and improves immunotherapy response.

**Table S1.** Clinical information of patients.
**Table S2.** Differentially expressed genes (high vs low immune scores).
**Table S3.** Survival analysis of immune‐related prognostic genes.
**Table S4.** Heatmaps showed cIPG expressions and survival times of LUAD cases.
**Table S5.** SNV analysis results.
**Table S6.** CNV analysis results.
**Table S7.** Methylation analysis results.

## Data Availability

The RNA‐seq data reported in this paper have been deposited in the OMIX, China National Center for Bioinformation/Beijing Institute of Genomics, Chinese Academy of Sciences (https://ngdc.cncb.ac.cn/omix: accession no. OMIX005058). The data and scripts used are saved in GitHub https://github.com/argo-bio/LTBR-acts-as-a-novel-immune-checkpoint-of-tumor-associated-macrophages-for-cancer-immunotherapy.git. Supporting Information (figures, tables, scripts, graphical abstract, slides, videos, Chinese translated version, and update materials) can be found in the online DOI or iMeta Science http://www.imeta.science/.
