# Supplementary material for: LTBR acts as a novel immune checkpoint of tumor‐associated macrophages for cancer immunotherapy
Source: Imeta. 2024 Sep 4;3(5):e233. doi: 10.1002/imt2.233 (PMC11487550; doi:10.1002/imt2.233)
Supplement: Supplementary file 1 — Figure S1. Immune scores are utilized to screen out immune‐related genes in LUAD. Figure S2. The immune‐related prognostic genes were screened in TCGA and GEO database. Figure S3. iMOS analysis reveals LTBR as a potential immune‐related mediator in LUAD. Figure S4. iMOS analysis reveals LTBR as a potential TAMs immune checkpoint in LUAD. Figure S5. LTBR is associated with LUAD stages, clinical prognosis and immunotherapy failure. Figure S6. LTBR maintains TAMs immunosuppressive activity and M2 phenotype. Figure S7. LTBR maintains TAM immunosuppressive behavior and M2 phenotype by noncanonical NF‐κB signalling and Wnt/β‐catenin signaling. Figure S8. Knockout of LTBR in TAMs impedes tumor growth via disrupting TAM immunosuppressive activities and M2 phenotype. Figure S9. TAM‐targeted delivery of LTBR siRNA disrupts TAM immunosuppressive ability and improves immunotherapy response. Figure S10. TAM‐targeted delivery of LTBR siRNA disrupts TAM immunosuppressive ability and improves immunotherapy response. [file IMT2-3-e233-s002.docx]

**Supporting information to**

**LTBR acts as a novel immune checkpoint of tumor-associated macrophages for cancer immunotherapy**

**Running title**: LTBR as a novel myeloid checkpoint for cancer immunotherapy

Liang Wang^1,#^, Jieyi Fan^2,#^, Sifan Wu^1,#^, Shilin Cheng^1,#^, Junlong Zhao^1^, Fan Fan^1^, Chunchen Gao^1^, Rong Qiao^3^, Qiqi Sheng^1^, Yiyang Hu^1^, Yong Zhang^4^, Pengjun Liu^1^, Zhe Jiao^1^, Tiaoxia Wei^1^, Jie Lei^5^, Yan Chen^3,*^, Hongyan Qin^1,*^

1State Key Laboratory of Holistic Integrative Management, Department of Medical Genetics and Developmental Biology, Fourth Military Medical University, Xi’an, 710032, China

2Department of Aerospace Medicine, Fourth Military Medical University, Xi'an,710032, China

3Department of Clinical Oncology, Xijing Hospital, Fourth Military Medical University, Xi'an,710032, China

4Department of Pulmonary Medicine, Xijing Hospital, Fourth Military Medical University, Xi'an,710032, China

5Department of Thoracic Surgery, Tangdu Hospital, Fourth Military Medical University, Xi’an, 710032, China

^#^These authors contributed equally: Liang Wang, Jieyi Fan, Sifan Wu and Shilin Cheng

^*^Correspondence: [hyqin@fmmu.edu.cn](mailto:hyqin@fmmu.edu.cn) (Hongyan Qin), [chenyanfmmu@163.com](mailto:chenyanfmmu@163.com) (Yan Chen)

**Supplementary Figures:**

**
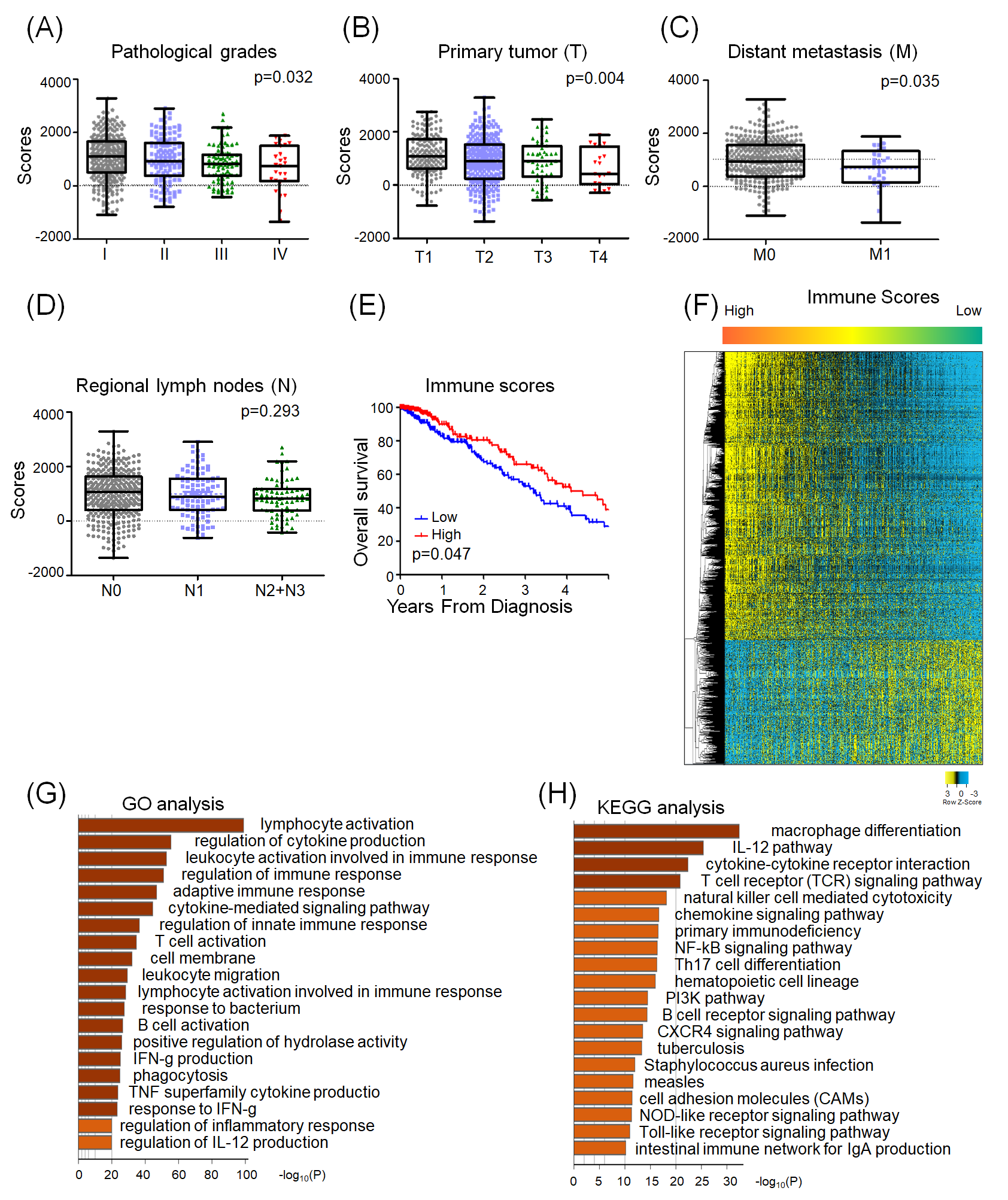
**

**Figure S1. Immune scores are utilized to screen out immune-related genes in LUAD.**

(A) Box & whisker plot shows the distribution of immune scores in LUAD different pathological stages. (B-D) Box & whisker plot shows the distribution of immune scores in LUAD different T (tumor) stages (B), M (metastasis) stages (C), N (lymph node) stages (D). The *p* value in A–D was indicated by one-way ANOVA test. (E) LUAD patients were divided into two groups by their immune scores: high scores and low scores, which is shown by Kaplan-Meier survival plot. The *p* value was indicated by the log‐rank test. (F) Based on LUAD immune scores, immune-related differentially expressed genes (DEGs) were screened out (*p* < 0.05, fold change > 1.5) and displayed by the heatmap via the method of average linkage and Pearson distance measurement. LUAD cases with higher scores were colored by red, lower scores were colored by green in the top color bar. Genes with higher expression were colored by yellow, lower expression were colored by blue, genes with same expression level were colored by black. (G and H) GO and KEGG analysis of the DEGs were performed by Metascape website, and the top 20 significant GO (G) and KEGG (H) terms were shown.


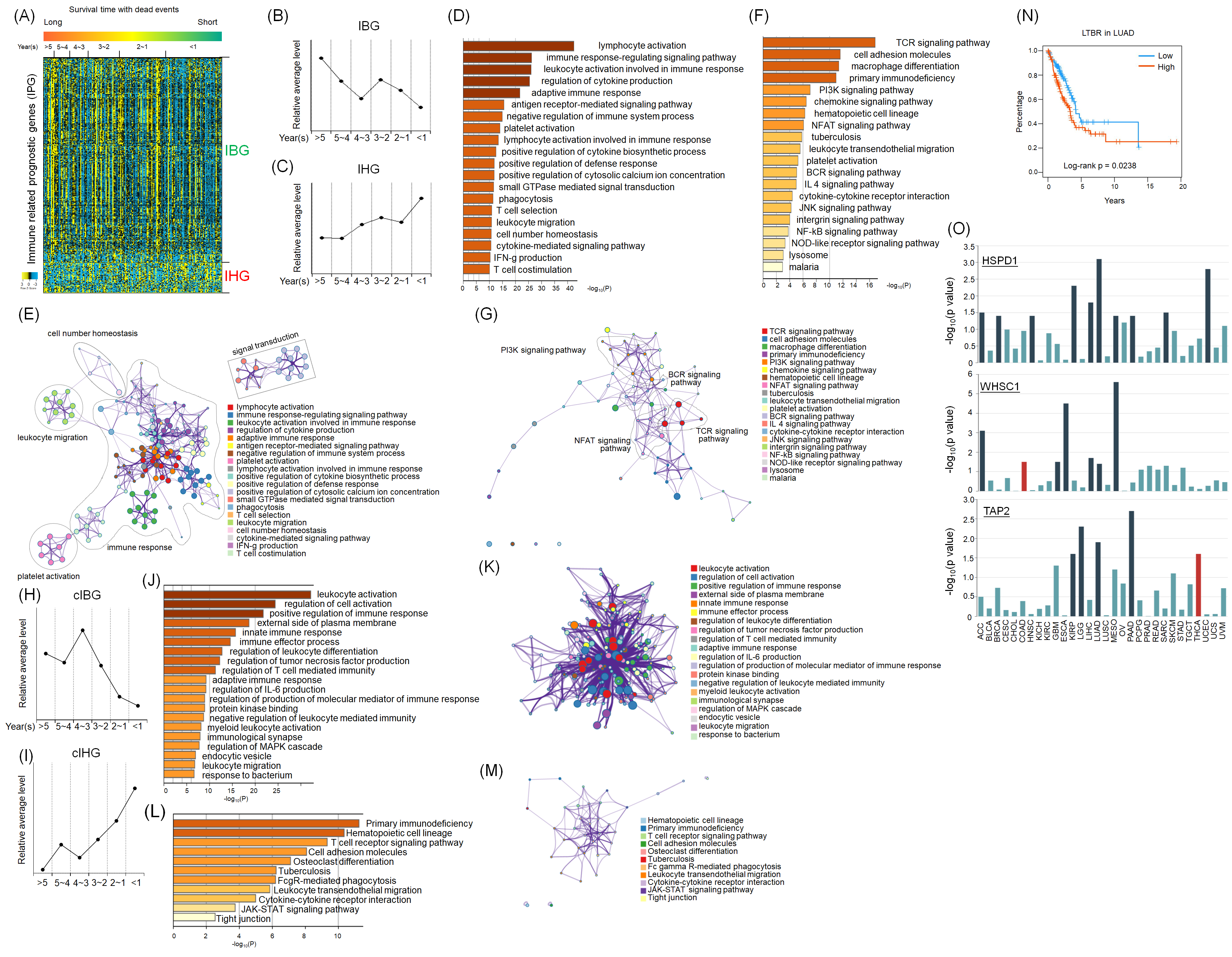


**Figure S2. The immune-related prognostic genes were screened in TCGA and GEO database.**

(A) The association between LUAD survival time and the expression of immune-related genes were analyzed by Kaplan-Meier method in order to obtain immune related prognostic genes (IPG) including immune related beneficial genes (IBG, higher expression associated with longer survival) and harmful gene (IHG, higher expression associated with shorter survival), *p* < 0.05 by the log-rank test. Heatmaps showed IPG expressions (rows) and survival times of LUAD cases. LUAD cases with longer survival time were colored by red, shorter survival time were colored by green in the top color bar. Genes with higher expression were colored by yellow, lower expression were colored by blue, genes with same expression level were colored by black. (B and C) The trendgram showed the relative average level of IBG (B) and IHG (C) accompanied by different survival time. (D-G) GO and KEGG analysis of the IPG were performed by Metascape website, and the top 20 significant GO (D) and KEGG (F) terms were shown. The significant GO (E) and KEGG (G) clusters of IPG were colored by cluster terms. The network diagram showed the subset of cluster terms from GO (D) and KEGG (F) clusters. Each term was displayed by a node, and its size is proportional to the quantity of input genes that belong to this term. The node color depicts the identity of the cluster (on the right legend) that the term belongs to. Terms with a similarity score > 0.3 were connected by an edge, whose weight represents the similarity score. (H and I) The trendgram showed the relative average level of credible IBG (cIBG) (H) and credible IHG (cIHG) (I) accompanied by the survival time of LUAD patients. (J-M) GO (J) and KEGG (L) analysis of the cIPG were performed by Metascape website, and the significant GO (K) and KEGG (M) clusters of cIPG were colored by cluster terms. The network diagram showed the subset of cluster and each term was displayed by a node, and its size is proportional to the quantity of input genes that belong to this term. The node color depicts the identity of the cluster (on the right legend) that the term belongs to. Terms with a similarity score > 0.3 were connected by an edge, whose weight represents the similarity score. (N) Kaplan-Meier plot showed the high expression of LTBR were associated with poor survival, *p* value by log-rank test. (O) The histogram showed pan-cancer survival analysis of HSPD1, WHSC1 and TAP2, dark blue column indicating that higher expression of the gene correlates with shorter survival (*p* < 0.05), red column indicating that higher expression of the gene correlates with longer survival (*p* < 0.05), while light blue indicating no significance by log-rank test.


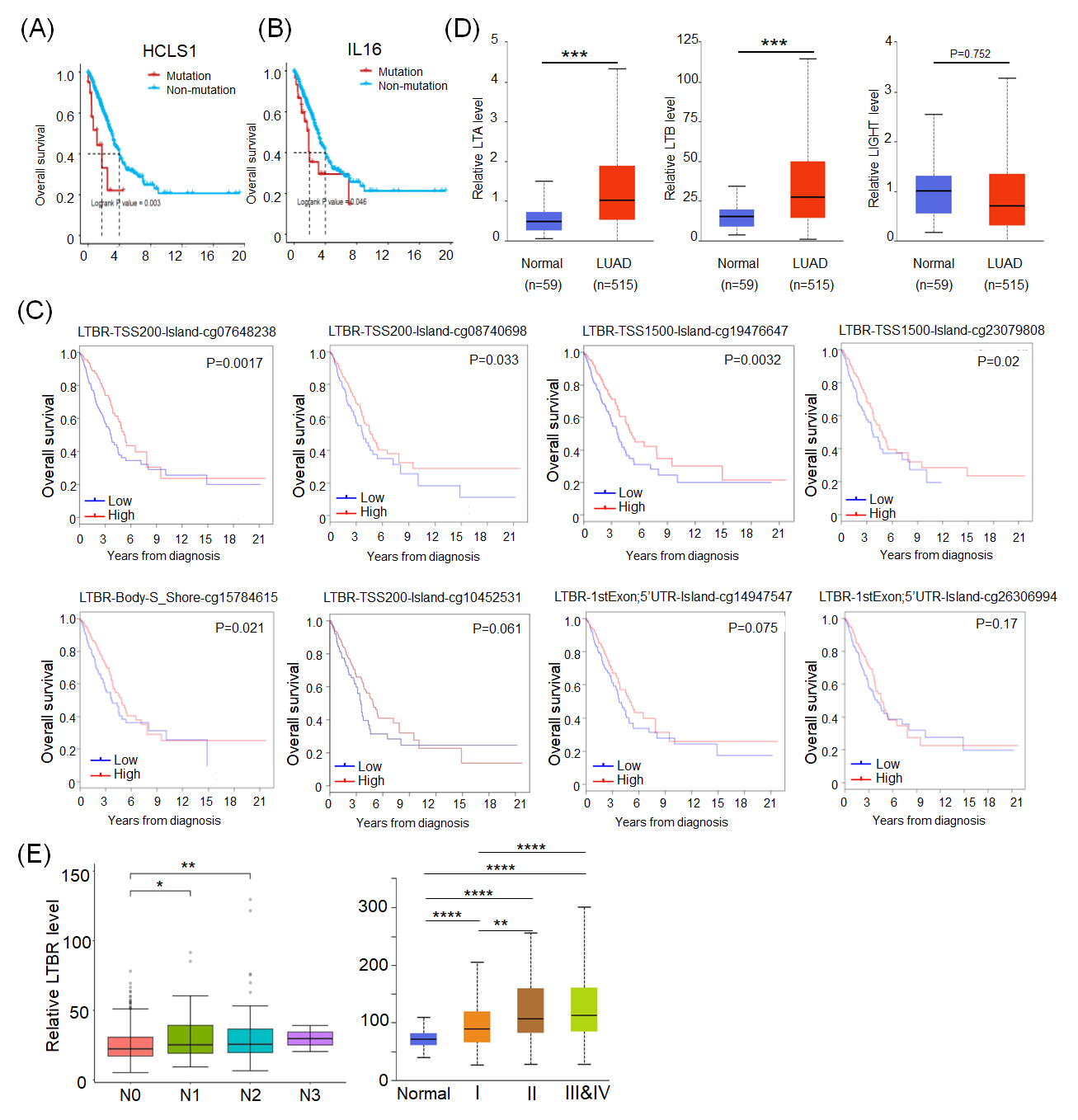


**Figure S3. iMOS analysis reveals LTBR as a potential immune-related mediator in LUAD.**

(A and B) Kaplan-Meier plot showed mutated HCLS1 (A) and IL16 (B) were associated with poor survival, *p* < 0.05 by log-rank test. (C) Kaplan-Meier plot showed the association between LUAD overall survival and the methylation level of LTBR. The *p* value was calculated via log-rank test. (D) The relative level of LTA, LTB and LIGHT in primary LUAD tissues (n = 515) and normal lung tissues (n = 59) was analyzed by using TCGA database. (E) The relative level of LTBR in different N stages and pathological grades was analyzed using TCGA database. Data are shown as mean ± s.e.m. *, *p* < 0.05; **, *p* < 0.01; ***, *p* < 0.001; ****, *p* < 0.0001 by unpaired student’s t-test (D) or one way ANOVA with Tukey's multiple comparison test (E).


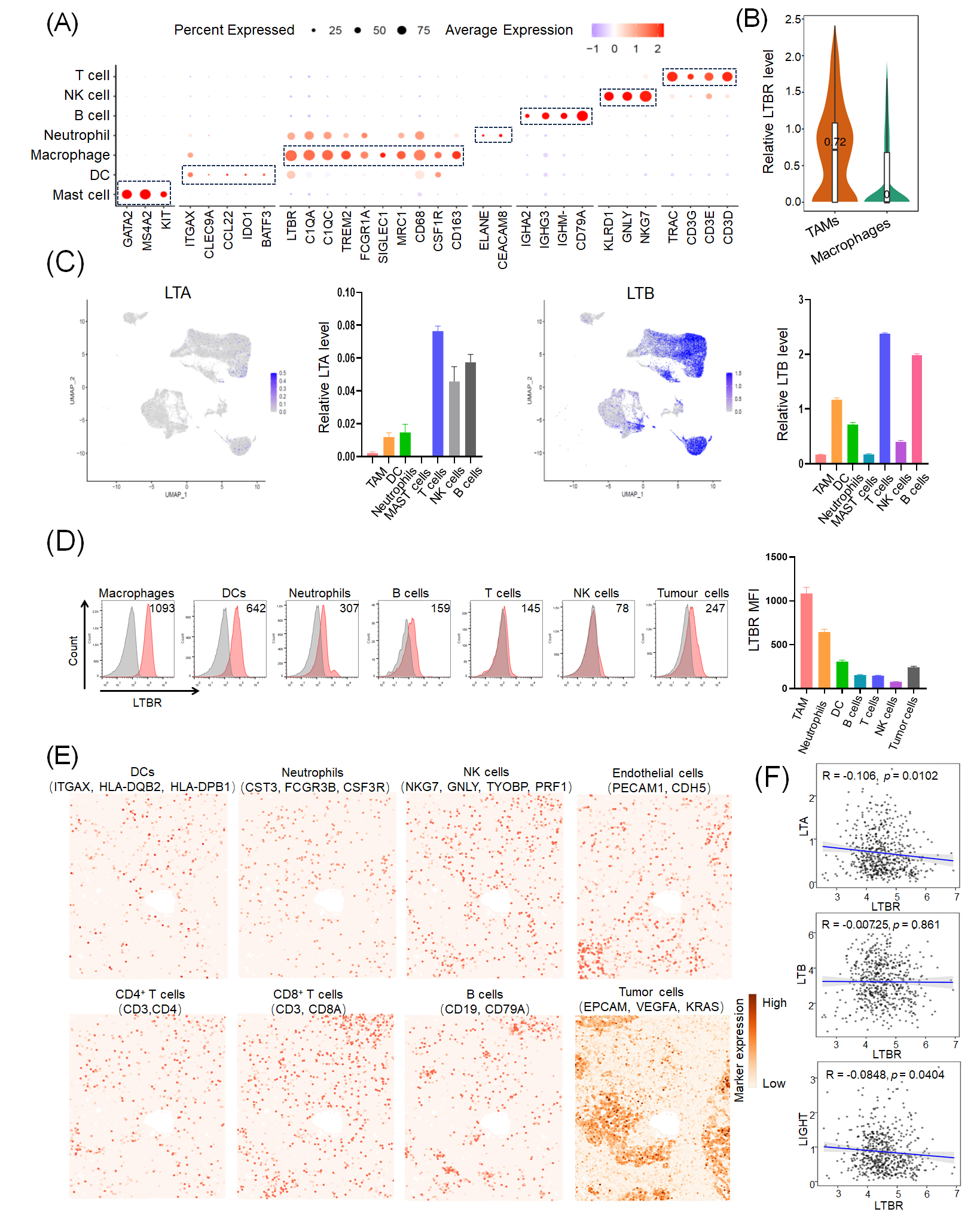


**Figure S4. iMOS analysis reveals LTBR as a potential TAM immune checkpoint in LUAD**.

(A) Bubble heatmap showed expression levels of selected signature genes in immune cells of human LUAD. Dot size indicated fraction of expressing cells, colored based on normalized expression levels. (B) Using human LUAD[1] (GSE131907) and normal lung scRNA-seq data (GSE134355), violin plot showed the expression of LTBR between TAMs and macrophages derived from LUAD and normal lung tissue, respectively. (C) UMAP plots and histogram showing the expression distribution of LTA and LTB in human LUAD immune cells. (D) The MFI of LTBR among tumor and immune cells was measured by FACS (n = 3). (E) Spatial scRNA-seq analysis showed the spatial location of LUAD-infiltrated immune cells. (F) The correlation between LTBR expression and its ligand expression was tested via Pearson correlation analysis. Data are shown as mean ± s.e.m. *, *p* < 0.05; **, *p* < 0.01; ***, *p* < 0.001; ****, *p* < 0.0001 by unpaired student’s t-test (B).


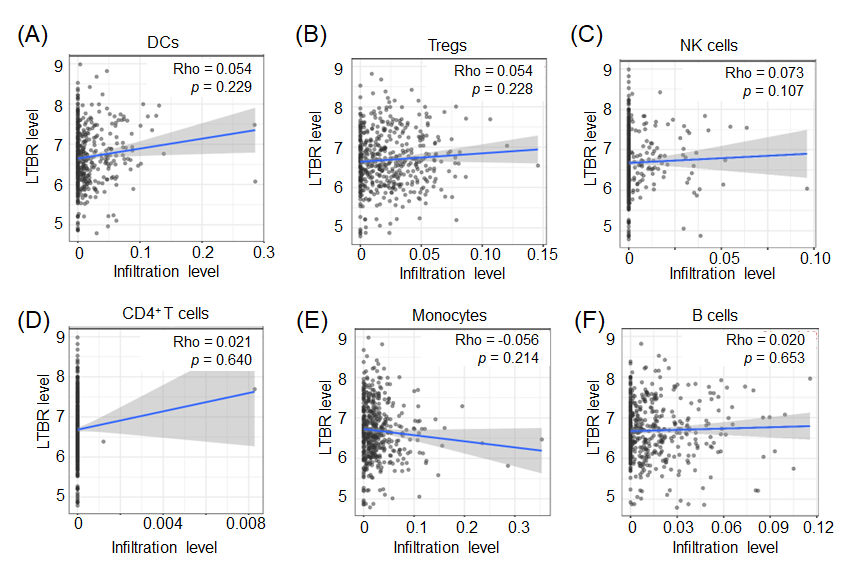


**Figure S5. LTBR is associated with LUAD stages, clinical prognosis and immunotherapy failure**

(A-F) The correlation between LTBR expression and the infiltration of indicated immune cells was analyzed by using TIMER2.0 website.


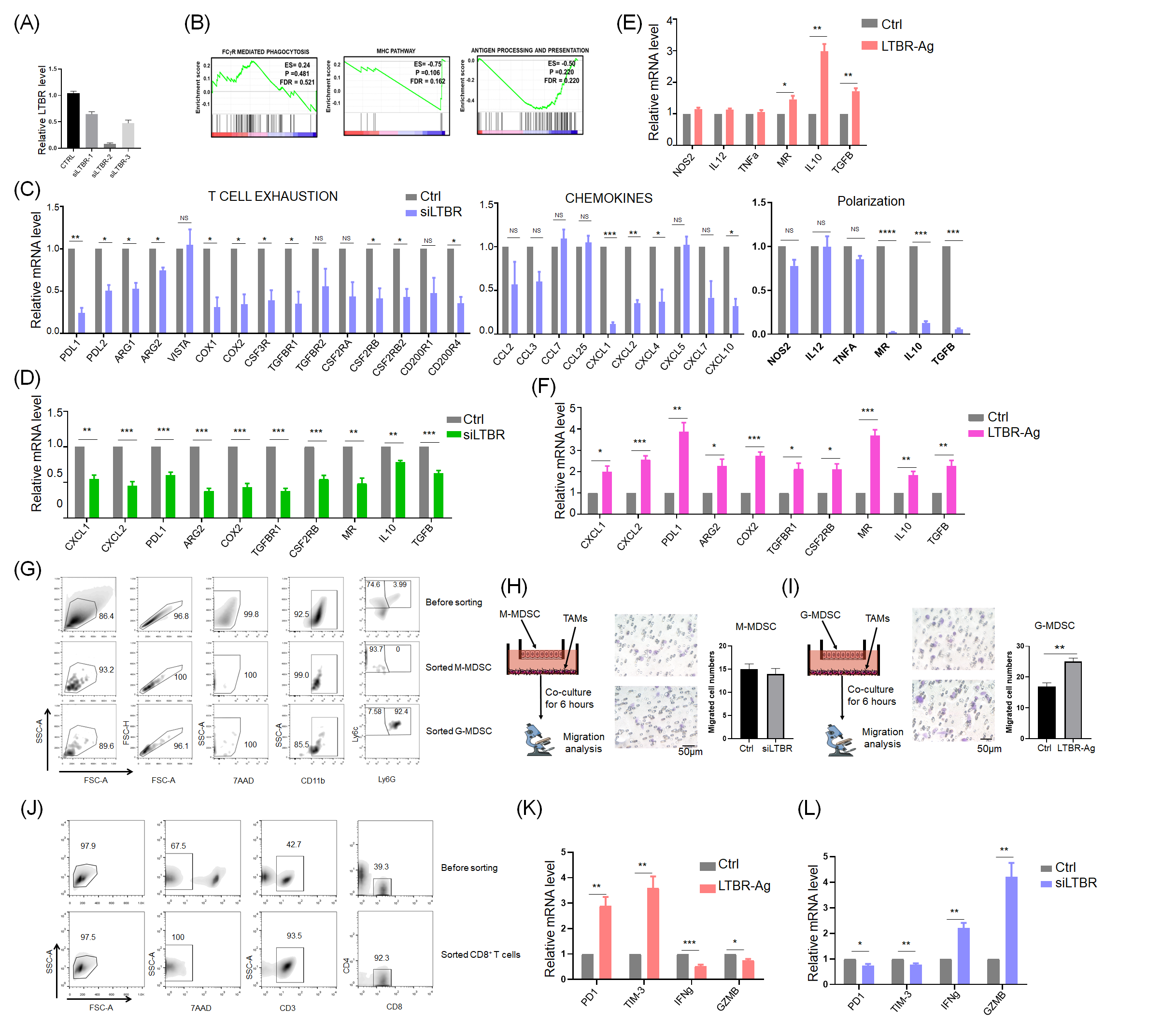


**Figure S6. LTBR maintains TAM immunosuppressive activity and M2 phenotype.**

(A) The knockdown efficiency of siRNA against LTBR in macrophages was measured by qRT-PCR (n = 3). (B) RNA-sequencing data of TAMs treated with control siRNA (Ctrl) and LTBR siRNA (siLTBR) was utilized for gene set enrichment analysis (GSEA) of FCγR mediated phagocytosis, MHC pathway as well as antigen processing and presentation. (C) After transfection of siLTBR or Ctrl in TAMs, the mRNA level of the indicated genes was measured by qRT-PCR (n = 3). (D) After transfection of siLTBR or Ctrl in RAW264.7 cells, the mRNA level of the indicated genes was measured by qRT-PCR (n = 3). (E) After activation of LTBR in TAMs by agonistic LTBR antibodies (LTBR-Ag), the expression of genes involved in M1 and M2 polarization was assayed by qRT-PCR (n = 3). (F) After activation of LTBR in RAW264.7 cells by agonistic LTBR antibodies (LTBR-Ag), the expression of indicated genes was assayed by qRT-PCR (n = 3). (G) The gating strategy of M-MDSC (7AAD^-^CD11b^+^Ly6c^+^Ly6G^-^) and G-MDSC (7AAD^-^CD11b^+^Ly6c^low^Ly6G^+^) from LLC-bearing mice before sorting. And then the cell purity was analyzed after sorting. (H) Coculture of TAMs (down) and sorted M-MDSC (up) by transwell system, the migration of M-MDSC was measured by microscope (n = 3). (I) Coculture of TAMs (down) and sorted G-MDSC (up) by transwell system, the migration of G-MDSC was measured by microscope (n = 3). (J) The gating strategy of CD8^+^ T cells (7AAD^-^CD3^+^CD4^-^CD8^+^) from the lymph node and spleen of wild type mice. And then the cell purity was analyzed after sorting. (K) TAMs were treated with agonistic LTBR antibodies (LTBR-Ag) or control IgG (Ctrl), and then co-cultured with CD8^+^ T cells. After that, the expression of the indicated genes in CD8^+^ T cells was assayed by qRT-PCR (n = 3). (L) TAMs were transfected with siLTBR or control siRNA (Ctrl), and then co-cultured with CD8^+^ T cells. After that, the expression of the indicated genes in CD8^+^ T cells was assayed by qRT-PCR (n = 3). Data are shown as mean ± s.e.m. *, *p* < 0.05; **, *p* < 0.01; ***, *p* < 0.001; ****, *p* < 0.0001 by unpaired student’s t-test.


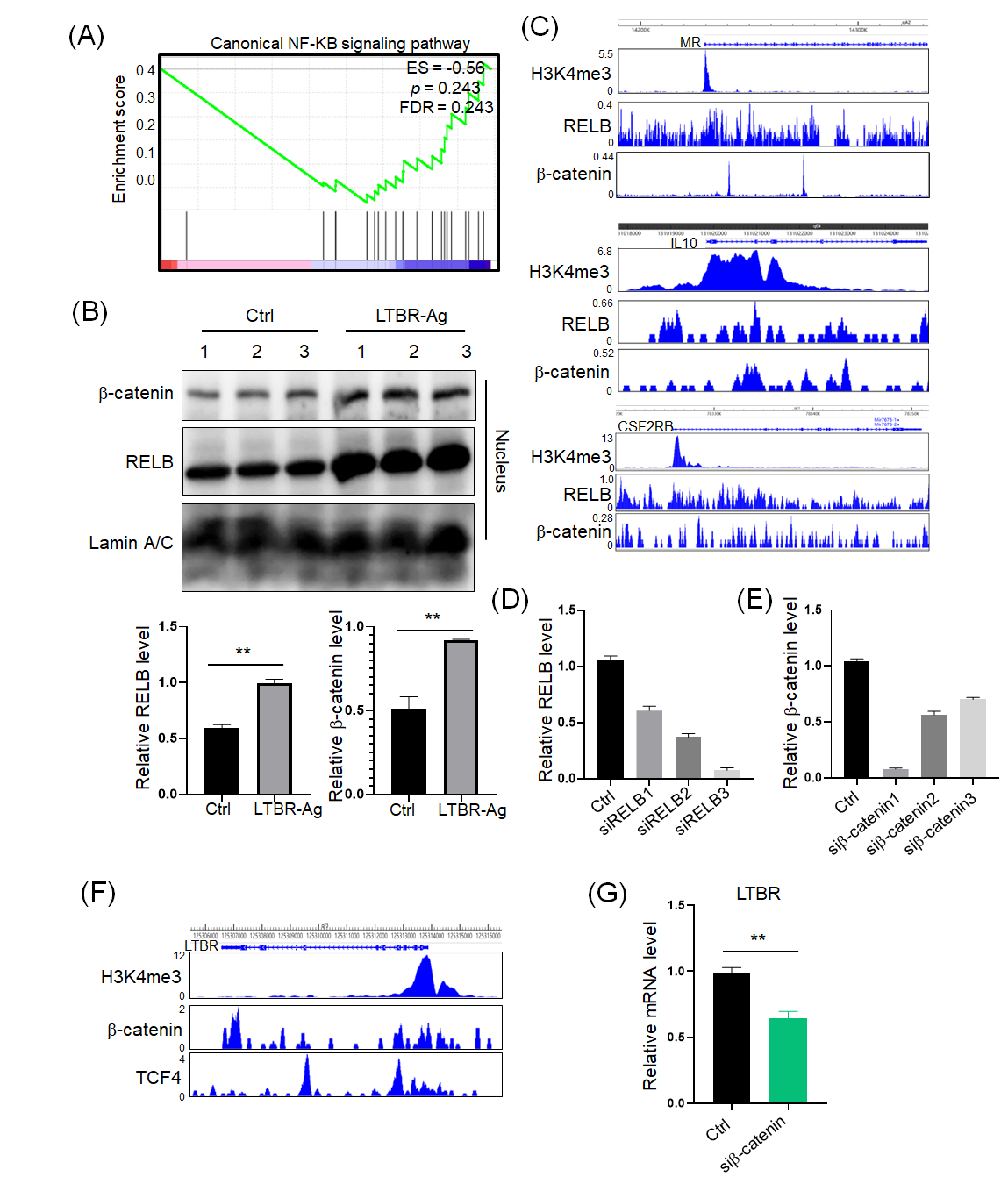


**Figure S7. LTBR maintained TAM immunosuppressive behavior and M2 phenotype by non-canonical NF-kB signalling and Wnt/b-catenin signaling**

(A) RNA-sequencing data of TAMs treated with control siRNA (Ctrl) and LTBR siRNA (siLTBR) was utilized for gene set enrichment analysis (GSEA) of canonical NF-kB signaling. (B) TAMs were treated with agonistic LTBR antibodies (LTBR-Ag) or control IgG (Ctrl), the nuclear protein level of indicated genes was detected by Western blot and quantitatively compared (n = 3). (C) ChIP-seq data from the Cistrome Project were utilized to analyze potential sites bound by RELB, β-catenin and H3K4me3 on the promoter of MR, IL10 and CSF2RB. (D and E) The knockdown efficiency of RELB (D) and β-catenin (E) siRNA was measured by qRT-PCR (n = 3). (F) ChIP-seq data from the Cistrome Project were utilized to analyze the binding level of β-catenin and H3K4me3 on the promoter of LTBR. (G) After transfection of siβ-catenin or Ctrl in TAMs, the mRNA level of LTBR was measured by qRT-PCR (n = 3). Data are shown as mean ± s.e.m. *, *p* < 0.05; **, *p* < 0.01; ***, *p* < 0.001 by unpaired student’s t-test.


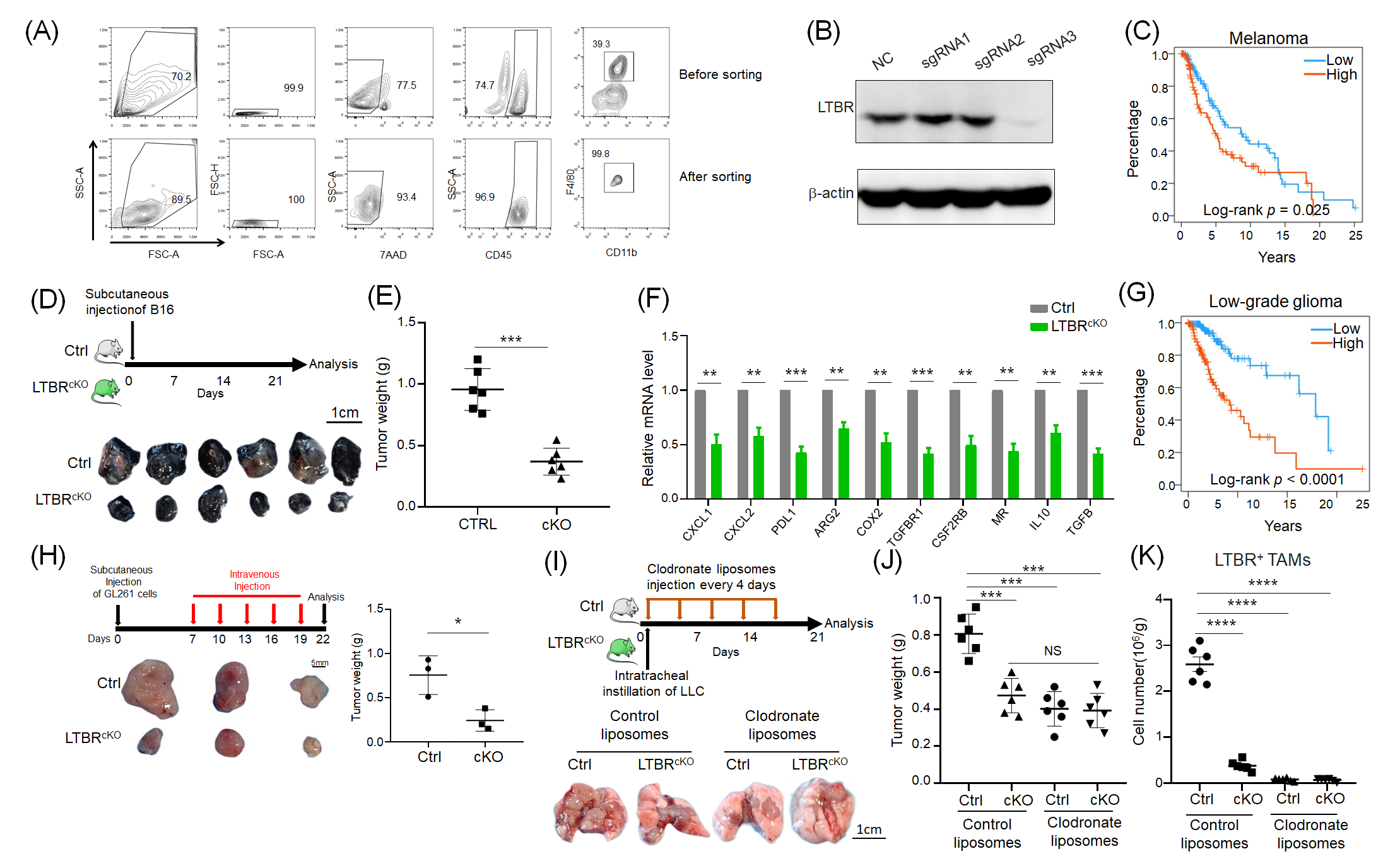


**Figure S8. Knockout of LTBR in TAMs impedes tumor growth via disrupting TAM immunosuppressive activities and M2 phenotype.**

(A) The gating strategy of TAMs (7AAD^-^CD45^+^CD11b^+^F4/80^+^) from LLC-bearing mice before sorting. And then the cell purity was analyzed after sorting. (B) Macrophage-specific Cas9-expressed tumor-bearing mice were intravenously injected with lentivirus carrying sgRNA for LTBR and the control (1×10^8^ particles per mouse). After that, TAMs were sorted as (A), and the protein level of LTBR in TAMs were analyzed by western blotting. (C) Kaplan-Meier plot showed the high expression of LTBR were associated with poor survival in melanoma, *p* value by log-rank test. (D) The melanoma mouse model was established by subcutaneous injection of B16 cells in macrophage-specific LTBR knockout (LTBR^cKO^) mice and the control (Ctrl) mice. Three weeks after B16 cells inoculation, the tumors were dissected and photographed. (E) The tumor weight of mice from (D) were measured and compared (n = 6). (F) Three weeks after the establishment of melanoma model, TAMs from macrophage-specific LTBR knockout (LTBR^cKO^) mice and the control (Ctrl) mice were sorted. The expression of the indicated genes in these sorted TAMs was measured by qRT-PCR (n = 3). (G) Kaplan-Meier plot showed the high expression of LTBR were associated with poor survival in low-grade glioma patients, *p* value by log-rank test. (H) Glioma mouse model was established in LTBR^cKO^ mice and Ctrl mice. Three weeks after inoculation, the tumor weight was analyzed. (I) Orthotopic lung cancer mouse model was established in LTBR^cKO^ mice and Ctrl mice, and then treated with control liposomes or clodronate liposomes every 4 days for five times. Three weeks after LLC inoculation, the tumors were dissected and photographed. (J) The tumor weight of mice from (I) were measured and compared (n = 6). (K) The infiltration of LTBR^+^ TAMs from (I) was analyzed by flow cytometry (n = 6). Data are shown as mean ± s.e.m. *, *p* < 0.05; **, *p* < 0.01; ***, *p* < 0.001; ****, *p* < 0.0001 by unpaired student’s t-test (E, F, and H) or one way ANOVA with Tukey's multiple comparison test (J and K).


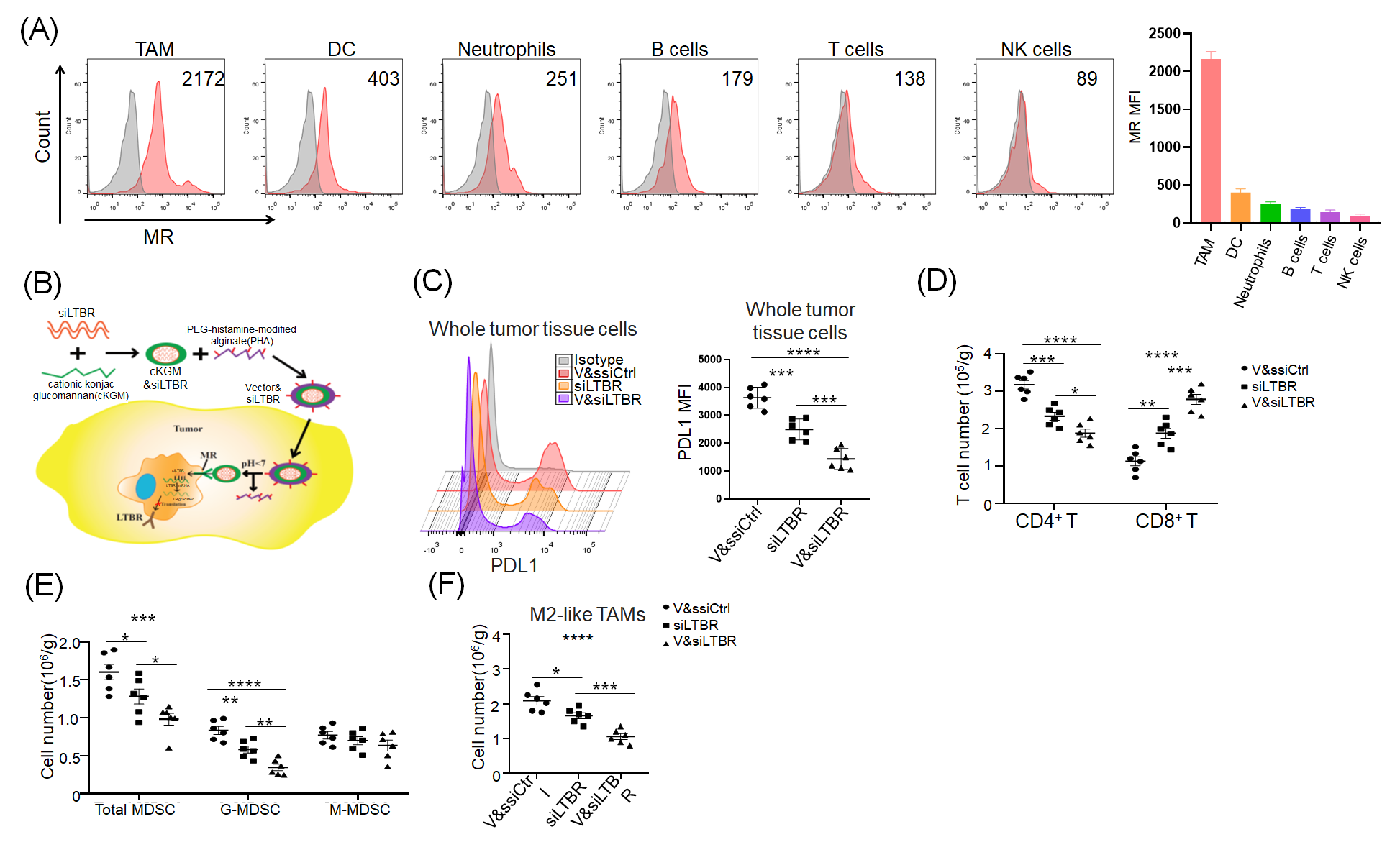


**Figure S9. TAM-targeted delivery of LTBR siRNA disrupts TAM immunosuppressive ability and improves immunotherapy response.**

(A) In lung cancer mouse model, the MFI of MR among different immune cells was analyzed by FACS (n = 3). (B) The schematic diagram of TAM-targeted siLTBR delivery system. (C) The PD-L1 MFI of whole tumor tissue cells with different treatments was measured by FACS (n = 6). (D-F) Orthotopic lung cancer model was established by intratracheally instillation of Lewis lung carcinoma (LLC) cells. One week after instillation, tumor-bearing mice were intravenously injected with V&siCtrl, siLTBR, V&siLTBR once three days. After five treatments, the proportion of T cells (D), MDSC (E) and M2-like TAMs (F) was measured by FACS (n = 6). Data are shown as mean ± s.e.m. *, *p* < 0.05; **, *p* < 0.01; ***, *p* < 0.001; ****, *p* < 0.0001 using one way ANOVA with Tukey's multiple comparison test (C-F).


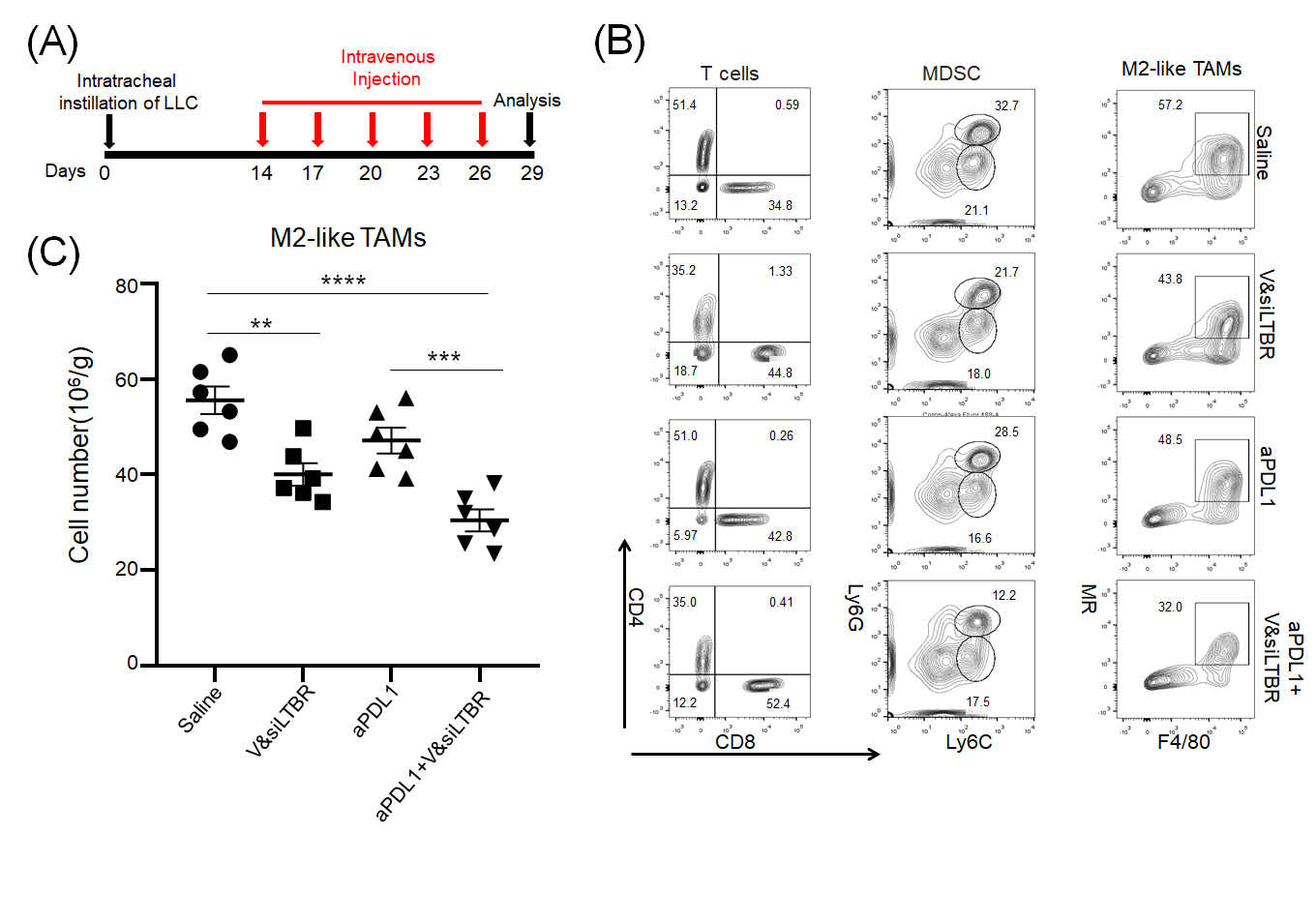


**Figure S10. TAM-targeted delivery of LTBR siRNA disrupts TAM immunosuppressive ability and improves immunotherapy response.**

(A) After the establishment of orthotopic lung cancer model, tumor-bearing mice were treated with saline, V&siLTBR, PDL1 antibody (aPDL1), V&siLTBR+aPDL1 every three days for five times. (B and C) After different treatment as (A), the proportion of T cells, MDSC and M2-like TAMs was measured by FACS (n = 6). Data are shown as mean ± s.e.m. *, *p* < 0.05; **, *p* < 0.01; ***, *p* < 0.001; ****, *p* < 0.0001 using one way ANOVA with Tukey's multiple comparison test (C).

**2. Supplementary resources table:**

| REAGENT or RESOURCE | SOURCE | IDENTIFIER |
| --- | --- | --- |
| **Antibodies** | | |
| LTBR | Affinity | Cat: #DF7292 |
| PE-LTBR(5G11) | Biolegend | Cat: #134403 |
| LTBR agonistic antibody (3C8) | Abcam | Cat: #ab171241 |
| b-catenin(E247) | Abcam | Cat: #ab32572 |
| RelB (D7D7W) | Cell Signaling Technology | Cat: #10544S |
| CD68(514H12) | Thermo Fisher | Cat: #MA1-80133 |
| Lamin A/C(4C11) | Cell Signaling Technology | Cat: # 4777 |
| β-actin(AC-15) | Sigma | Cat: #A1978 |
| COX2 (D5H5) | Cell Signaling Technology | Cat: # 12282 |
| ARG2 | Abcam | Cat: # Ab264066 |
| CSF2RB | Thermo Fisher | Cat: #PA5-115055 |
| TGFBR1 | Proteintech | Cat: #30117-1-AP |
| PE IL-12(C17.8) | eBioscience | Cat: #127123 |
| PE NOS2(CXNFT) | eBioscience | Cat: #12-5920 |
| PE PDL-1 (MIH5) | ebioscience | Cat: #125982 |
| APC IL-10(JES5-16E3) | eBioscience | Cat: #177101 |
| APC-CD11b(M1/70) | Biolegend | Cat: #101212; RRID: AB_312795 |
| Alexa488-F4/80(BM8) | Biolegend | Cat: #123120; RRID: AB_893479 |
| Biotin-Ly6G(1A8) | Biolegend | Cat: #127603; RRID: AB_1186105 |
| APC-Ly6C(HK1.4) | Biolegend | Cat: #128015; RRID: AB_1732087 |
| PE-avidin | eBioscience | Cat: #12-4317-87 |
| BV421-CD45(30-F11) | BD | Cat: #563890 |
| Biotin-MHC II(KH74) | BD | Cat: #553607 |
| PE-CD4(RM4-5) | BD | Cat: #553049 |
| APC-CD8(53-6.7) | BD | Cat: #553030 |
| FITC-CD3(17A2) | BD | Cat: #555274 |
| APC-B220(RA3-6B2) | Biolegend | Cat: #103211 |
| Biotin-NK1.1(PK136) | Biolegend | Cat: #108703 |
| Goat anti-Rabbit IgG, HRP | ZHAUNGZHI | Cat: #EK020 |
| Goat anti-Mouse IgG, HRP | BOSHIDE | Cat: #BA1050 |
| **Biological Samples** | | |
| Lung cancer tissue samples | Department of Thoracic Surgery, Tangdu Hospital | N/A |
| **Chemicals, Peptides, and Recombinant Proteins** | | |
| 7-AAD | eBioscience | Cat: #00-6993-50 |
| D-Luciferin,Potassium Salt D | Yeasen | Cat: #40902ES02 |
| Hoechst 33342 | Thermo Fisher | Cat: #62249 |
| Crystal violet | Beyotime | Cat: #C0121 |
| Polyethylene glycol | Sangon | Cat: #25322-68-3 |
| D-Histidine | Sangon | Cat: #351-50-8 |
| Dextran | Sangon | Cat: #9004-54-0 |
| type V collagenase | Stem cell | Cat: #07430 |
| DMEM | Gibco | Cat: #11995065 |
| L-glutamine | Gibco | Cat: #A37736IP |
| fetal bovine serum | Gibco | Cat: #26400044 |
| M-CSF | SinoBio | Cat: #51112-MNAH |
| GM-CSF | SinoBio | Cat: #51048-MNAH |
| IL-6 | SinoBio | Cat: #50136-MNAE |
| Lipofectamine 2000 | Invitrogen | Cat: #11668500 |
| E. coli DNA polymerase I | NEB | Cat: #M0209 |
| RNase H | NEB | Cat: #M0297 |
| heat-labile UDG enzyme | NEB | Cat: #M0280 |
| Trizol reagent | Invitrogen | Cat: #15596018 |
| **Critical Commercial Assays** | | |
| SuperScript™ II Reverse Transcriptase | Invitrogen | Cat: #18064014 |
| Dynabeads Oligo (dT)25 | Invitrogen | Cat: #61002 |
| CFSE | Sigma | Cat: # 21888 |
| Magnesium RNA Fragmentation Module | NEB | Cat: #E6150 |
| dUTP Solution | Thermo Fisher | Cat: #R0133 |
| HiScriptII Q RT SuperMix Reagent Kit | Vazyme | Cat: #R222 |
| ChamQ SYBR qPCR Master Mix Kit | Vazyme | Cat: #Q341 |
| Cytoplasmic and Nuclear Protein Extraction Kit | Beyotime | Cat: #P0028 |
| CD8a^+^ T cell Isolation Kit | Miltenyi | Cat: #130-104-075 |
| IL-10 Mouse ELISA Kit | Invitrogen | 88-7105-88 |
| TGFβ1 Mouse ELISA Kit | Invitrogen | Cat: #BMS608-4 |
| CXCL1 Mouse ELISA Kit | Invitrogen | Cat: #EMCXCL1 |
| CXCL2 Mouse ELISA Kit | Invitrogen | Cat: #EMCXCL2 |
| IL-12 Mouse ELISA Kit | Invitrogen | Cat: #BMS616 |
| CCL2 Mouse ELISA Kit | Invitrogen | Cat: #BMS6005 |
| IFN gamma Mouse ELISA Kit | Invitrogen | Cat: #88-7314-88 |
| ChIP assays kit | Merck Millipore | Cat: #17-371 |
| Clodronate liposomes | FormuMax | F70101C-A-10 |
| **Deposited Data** | | |
| RNA-sequence | This paper | OMIX005058 |
| Lung adenocarcinoma scRNA-seq data | Kim et al., 2020[2] | GSE131907 |
| **Experimental Models: Cell Lines** | | |
| Lewis lung carcinoma (LLC) cells | ATCC | Cat: # CRL-1642 |
| **Experimental Models: Organisms/Strains** | | |
| C57BL/6 mice | Animal Experiment Center of the Fourth Military Medical University | N/A |
| **Oligonucleotides** | | |
| mLTBR-f: 5’ - tactctgctcaagcggcac | This paper | N/A |
| mLTBR-r: 5’ - caggggactctgctgttgtag | This paper | N/A |
| mRELB-f: 5’- acaggtggtgaggagctgta | This paper | N/A |
| mRELB-r: 5’- tcgtagggtggcgttttgaa | This paper | N/A |
| mARG1-f: 5’-ctccaagccaaagtccttagag | This paper | N/A |
| mARG1-r: 5’-aggagctgtcattagggacatc | This paper | N/A |
| mARG2-f: 5’-cctccacgggcaaattccttg | This paper | N/A |
| mARG2-r: 5’-cccactgaacgaggatacaca | This paper | N/A |
| mTGFβR1 f: 5’-gcagctcctcatcgtgttg | This paper | N/A |
| mTGFβR1 r: 5’-cagctgactgcttttctgtagttg | This paper | N/A |
| mTGFβR2 f: 5’-aggaccatccatccactgaa | This paper | N/A |
| mTGFβR2 r: 5’-tcttccagagtgaagccgtg | This paper | N/A |
| mCSF2RB f: 5’-aagggcacatgagagctgac | This paper | N/A |
| mCSF2RB r: 5’-cggggttctgtattggtgcT | This paper | N/A |
| mNOS2-f: 5’-gagtgcgcacctcggtgtta | This paper | N/A |
| mNOS2-r: 5’-ccgtgtaagaatgctggaacg | This paper | N/A |
| mIL12-f: 5’-agcgacagatgtatgaagactca | This paper | N/A |
| mIL12-r: 5’-cagcctgtatgtgccctgtg | This paper | N/A |
| mTNFα-f: 5’-gtgctcccttcaaaacctgca | This paper | N/A |
| mTNFα-r: 5’-cccagccagaggatttcagc | This paper | N/A |
| mMR-f: 5’-cctgctcctgacactcaaaca | This paper | N/A |
| mMR-r: 5’-gctgcgagtcaccattgttc | This paper | N/A |
| mIL10-f: 5’-ccttggggaaagagtagatgtcc | This paper | N/A |
| mIL10-r: 5’-gcagagattggaggccttgtg | This paper | N/A |
| mCd200r1 f: 5’-tcagtggcttcagaaaatgcaa | This paper | N/A |
| mCd200r1 r: 5’-atggccgacaaagtaaggca | This paper | N/A |
| mCd200r4 f: 5’-tgtgtctgggtcaagttgtact | This paper | N/A |
| mCd200r4 r: 5’-ggaaggcaggtgtctgtgtt | This paper | N/A |
| mTGFβ-f: 5’-aacttgagggagaagtaggaatgg | This paper | N/A |
| mTGFβ-r: 5’-caggagggagaacagaaactcca | This paper | N/A |
| mCOX2-f: 5’-ttaagtggagccagggta-3’ | This paper | N/A |
| mCOX2-r: 5’-agtcagatcgcttggata-3’ | This paper | N/A |
| mActin-f: 5’-ttgaaaggagccagtgtg | This paper | N/A |
| mActin-r: 5’-ctatcgtgggcactcagaagt | This paper | N/A |
| mCXCL1-f: 5’-ctgggattcacctcaagaacatc | This paper | N/A |
| mCXCL1-r: 5’-cagggtcaaggcaagcctc | This paper | N/A |
| mCXCL2-f: 5’-gaagtcatagccactctcaagg | This paper | N/A |
| mCXCL2-r: 5’-cttccgttgagggacagc | This paper | N/A |
| mCXCL4 f: 5’-ctggaggtgatcaaggcagg | This paper | N/A |
| mCXCL4 r: 5’-atataggggtgcttgccggt | This paper | N/A |
| mCXCL7 f: 5’-tgcgctgcagatgtacgaat | This paper | N/A |
| mCXCL7 r: 5’-agcagctggtcagtaaccttc | This paper | N/A |
| mCXCL10 f: 5’-ccaagtgctgccgtcatttt | This paper | N/A |
| mCXCL10 r: 5’-ctcaacacgtgggcaggata | This paper | N/A |
| mCCL7 f: 5’-tcaccagtagtcggtgtccc | This paper | N/A |
| mCCL7 r: 5’-ctatagcctcctcgacccact | This paper | N/A |
| mCCL25 f: 5’-agttcactgatcccataggca | This paper | N/A |
| mCCL25 r: 5’-atccaggccccaacaaaaca | This paper | N/A |
| mCXCL5 f: 5’-tgccctacggtggaagtcat | This paper | N/A |
| mCXCL5 r: 5’-tgcgagtgcattccgctta | This paper | N/A |
| mCSF3R f: 5’-acgacggggctagaaagaga | This paper | N/A |
| mCSF3R r: 5’-gctcccagccctaccatttt | This paper | N/A |
| mCCL2 f: 5’-aggtgtcccaaagaagctgt | This paper | N/A |
| mCCL2 r: 5’-aagaccttagggcagatgcag | This paper | N/A |
| mCCL3 f: 5’-ccaagtcttctcagcgcca | This paper | N/A |
| mCCL3 r: 5’-ggaatcttccggctgtaggag | This paper | N/A |
| mPDL1 f: 5’- gacgcaggcgtttactgct | This paper | N/A |
| mPDL1 r: 5’- gcggtatggggcatgacttt | This paper | N/A |
| mPDL2 f: 5’- ctgccgatactgaacctgagc | This paper | N/A |
| mPDL2 r: 5’-gcggtcaaaatcgcactcc | This paper | N/A |
| mIFNγ f: 5’- atgaacgctacacactgcatc | This paper | N/A |
| mIFNγ r: 5’- ccatccttttgccagttcctc | This paper | N/A |
| mVISTA f: 5’- ggaaccctgctccttgctatt | This paper | N/A |
| mVISTA r: 5’- ttgtagatggtcacatcgtgc | This paper | N/A |
| mCSF2RA f: 5’- ctgctcttctccacgctactg | This paper | N/A |
| mCSF2RA r: 5’- gagactcgccggtgtatcc | This paper | N/A |
| mCSF2RB2 f: 5’- tccagccagatcgtgacct | This paper | N/A |
| mCSF2RB2 r: 5’- aatccccaagagatacactcca | This paper | N/A |
| LTBR siRNA1: 5’- gaguuugucuuugcgguautt | This paper | N/A |
| LTBR siRNA2: 5’- cuacucggauaccaucugutt | This paper | N/A |
| LTBR siRNA3: 5’- ccggcaacaucuacauauatt | This paper | N/A |
| RELB siRNA1: 5’- gugacugucaauguguucutt | This paper | N/A |
| RELB siRNA2: 5’- guguggacaagaagcgaaatt | This paper | N/A |
| RELB siRNA3: 5’- gcagcaacauguuucccaatt | This paper | N/A |
| b-catenin siRNA1: 5’- gcaccaugcagaauacaaatt | This paper | N/A |
| b-catenin siRNA2:5’-gcaguugucaauuugauuatt | This paper | N/A |
| b-catenin siRNA3: 5’-ccaggcgagtttgtctttgcggtat | This paper | N/A |
| PDL1 ChIP f: 5’-aagtcccctagcaggaagca | This paper | N/A |
| PDL1 ChIP r: 5’- ggaatttgcggttctgtccc | This paper | N/A |
| ARG2 ChIP f: 5’-tgatcggctgattggcaagt | This paper | N/A |
| ARG2 ChIP r: 5’- agagctcactgtctagcgga | This paper | N/A |
| TGFBR1 ChIP f: 5’-cttcgtagggcaatcctccc | This paper | N/A |
| TGFBR1 ChIP r: 5’- ccagactcacgaagcgagaa | This paper | N/A |
| TGFB ChIP f: 5’- gactgtcgggggcacttaat | This paper | N/A |
| TGFB ChIP r: 5’- gcagtagcagcggaaaagtc | This paper | N/A |
| CXCL1 ChIP f: 5’- tggcatatgacccctgaacg | This paper | N/A |
| CXCL1 ChIP r: 5’- acacgtgcgtgttgaccata | This paper | N/A |
| CXCL2 ChIP f: 5’-gcgatttgtgcttacacctgac | This paper | N/A |
| CXCL2 ChIP r: 5’- cgaggcacatcaggtacgat | This paper | N/A |
| IL10 ChIP f: 5’- cggttctggcctgacgatt | This paper | N/A |
| IL10 ChIP r: 5’- aaagatttgtccgtctgctttggg | This paper | N/A |
| MR ChIP f: 5’- cccgcagacttccaactagg | This paper | N/A |
| MR ChIP r: 5’- acagttgctgcactcacctt | This paper | N/A |
| CSF2RB ChIP f: 5’- gtcactcctggagatccggt | This paper | N/A |
| CSF2RB ChIP r: 5’-gcaatgcagacacgcaatg | This paper | N/A |
| COX2 ChIP f: 5’-сассассссстттgсcатaa | This paper | N/A |
| COX2 ChIP r: 5’-cttccccтcccgggatctaa | This paper | N/A |
| LTBR ChIP f: 5’- attcagggccagctgaactc | This paper | N/A |
| LTBR ChIP r: 5’- cgtgcatgggctcgtagtat | This paper | N/A |
| LTBR sgRNA1: 5’-ccacgacttcccaagcctgg | This paper | N/A |
| LTBR sgRNA2: 5’-agaccgggtggagtcctagg | This paper | N/A |
| LTBR sgRNA3: 5’- ccgcagggagaggaggcccg | This paper | N/A |
| **Software and Algorithms** | | |
| ImageJ | National Institutes of Health | https://imagej.nih.gov/ij/ |
| R (versions 3.6.3) | R Core Team | https://www.R-project.org |
| FlowJo vX.0.6 | FlowJo, LLC | https://www.flowjo.com/ |
| Image Pro Plus 5.1 | Media Cybernetics | http://www.mediacy.com/imageproplus |
| GraphPad Prism 8 | GraphPad Software | https://www.graphpad.com/scientificsoftware/prism/ |
| BLOCK-iT_ RNAi Designer | Thermo Fisher Scientific | http://rnaidesigner.thermofisher.com/rnaiexpress/ |
| GSEA v4.3.2 | Broad Institute, Inc., Massachusetts Institute of Technology, and Regents of the University of California[3] | https://www.gsea-msigdb.org/gsea/index.jsp |
| Metascape | Metascape Team[4] | http://metascape.org |
| TIMER 2.0 | Liu Lab[5] | http://timer.cistrome.org/ |
| TISIDB | Zhang Lab[6] | http://cis.hku.hk/TISIDB |
| Cistrome database | Liu Lab[7] | http://www.cistrome.org |

**REFERENCES**

1. Cheng, Sijin, Ziyi Li, Ranran Gao, Baocai Xing, Yunong Gao, Yu Yang, Shishang Qin, et al. 2021. “A pan-cancer single-cell transcriptional atlas of tumor infiltrating myeloid cells.” *Cell* 184: 792-809 e723. <https://doi.org/10.1016/j.cell.2021.01.010>

2. Kim, Nayoung, Hong Kwan Kim, Kyungjong Lee, Yourae Hong, Jong Ho Cho, Jung Won Choi, Jung-Il Lee, et al. 2020. “Single-cell RNA sequencing demonstrates the molecular and cellular reprogramming of metastatic lung adenocarcinoma.” *Nature communications* 11: 2285. <https://doi.org/10.1038/s41467-020-16164-1>

3. Subramanian, Aravind, Pablo Tamayo, Vamsi K Mootha, Sayan Mukherjee, Benjamin L Ebert, Michael A Gillette, Amanda Paulovich, et al. 2005. “Gene set enrichment analysis: a knowledge-based approach for interpreting genome-wide expression profiles.” *Proceedings of the National Academy of Sciences of the United States of America* 102: 15545-15550. <https://doi.org/10.1073/pnas.0506580102>

4. Zhou, Yingyao, Bin Zhou, Lars Pache, Max Chang, Alireza Hadj Khodabakhshi, Olga Tanaseichuk, Christopher Benner, Sumit K Chanda. 2019. “Metascape provides a biologist-oriented resource for the analysis of systems-level datasets.” *Nature communications* 10: 1523. <https://doi.org/10.1038/s41467-019-09234-6>

5. Li, Taiwen, Jingxin Fu, Zexian Zeng, David Cohen, Jing Li, Qianming Chen, Bo Li, X Shirley Liu. 2020. “TIMER2.0 for analysis of tumor-infiltrating immune cells.” *Nucleic acids research* 48: W509-W514. <https://doi.org/10.1093/nar/gkaa407>

6. Ru, Beibei, Ching Ngar Wong, Yin Tong, Jia Yi Zhong, Sophia Shek Wa Zhong, Wai Chung Wu, Ka Chi Chu, et al. 2019. “TISIDB: an integrated repository portal for tumor-immune system interactions.” *Bioinformatics* 35: 4200-4202. <https://doi.org/10.1093/bioinformatics/btz210>

7. Liu, Tao, Jorge A Ortiz, Len Taing, Clifford A Meyer, Bernett Lee, Yong Zhang, Hyunjin Shin, et al. 2011. “Cistrome: an integrative platform for transcriptional regulation studies.” *Genome biology* 12: R83. <https://doi.org/10.1186/gb-2011-12-8-r83>
